# Towards Nickel–NHC Fluoro Complexes—Synthesis of Imidazolium Fluorides and Their Reactions with Nickelocene

**DOI:** 10.3390/molecules29184493

**Published:** 2024-09-21

**Authors:** Siobhan S. Wills, Corinne Bailly, Michael J. Chetcuti

**Affiliations:** 1 School of Chemistry, University of New South Wales (UNSW), Sydney, NSW 2052, Australia; siobhan.wills@unsw.edu.au; 2 Fédération de Chimie Le Bel UAR2042, BP20296, 1 rue Blaise Pascal, 67008 Strasbourg, France; c.bailly@unistra.fr; 3 Organometallic Research Group (OMCAT), LIMA CNRS UMR 7042, ECPM, University of Strasbourg, 25 rue Becquerel, 67087 Strasbourg, France

**Keywords:** imidazolium fluorides, N-heterocyclic carbene, nickel, fluoro-complexes, H-bonding

## Abstract

While hundreds of complexes of the general formula [Ni(η^5^-C_5_H_5_)(NHC)(X)] exist (NHC = a N-heterocyclic carbene, X = Cl, Br, I), none is yet known with X = F. We attempted to prepare such a species by reacting nickelocene with imidazolium fluorides. Three imidazolium fluorides (ImH)^+^ F^−^ [Im = (*N*,*N*′-*bis-*(R)-imidazolium: **1a**, IMe, R = Me; **1b**, IMes, R = 2,4,6-trimethylphenyl; **1c**, IPr, R = 2,6-diisopropylphenyl)] were prepared and characterized by spectroscopic methods. In addition, the salts **1b** [(IMesH)^+^ F^−^] and **1c** [(IPrH)^+^ F^−^] were subjected single-crystal X-ray diffraction experiments. The reactions of these imidazolium fluorides with nickelocene did not lead to [Ni(η^5^-C_5_H_5_)(NHC)(F)] species. Instead, the reaction of **1a** [(IMeH)^+^ F^−^] and **1b** [(IMesH)^+^ F^−^] with nickelocene led to the salt **2** [Ni(η^5^-C_5_H_5_)(IMe)_2_]^+^ F^−^ and to the square planar complex **3a** *trans-*[NiF_2_(IMes)_2_] respectively. Both complexes were characterized spectroscopically and by single crystal X-ray diffraction. All four X-ray diffraction studies reveal hydrogen bonding and hydrogen interactions with the F atom or anion, and in some cases with solvent molecules of crystallization, and these phenomena are all discussed. Complex **2**, in particular, exhibited a wide range of interesting H-bonded interactions in the solid state. Complexes **2** and **3a** were tested as catalysts for Suzuki–Miyaura coupling but were not promising: complex **2** was inactive, and while **3a** did indeed catalyze the reaction, it gave widely diverging results owing to its instability in solution.

## 1. Introduction

Organofluorine compounds are key molecules in the pharmaceutical and agrochemical industries. In 2020, it was estimated that ≈20% of commercial pharmaceuticals are fluoro-pharmaceuticals, [1] including best-sellers such as the anti-cholesterol drug Lipitor, the anti-depressive Prozac (fluoxetine) and the antibiotic Cipro (ciprofloxacin), and the proportion has increased since then. Nevertheless, not many transition-metal catalysts are known that can introduce fluorine into organic molecules. Fluorine is the most electronegative element and (after He) the smallest. Established synthetic techniques for the introduction of halogens into organometallic compounds are not generally applicable to fluorine [2], as carbon forms its strongest single bond with fluorine [3], and this inert C–F bond makes oxidative addition of RF compounds to organometallic reagents more difficult. Fluoride-for-halide exchange reactions are thus more difficult; furthermore, the ease of reaction of F^−^ ions with glass [4] potentially complicates its reactions. For these reasons, fluorine organometallics are less common, and this area is “still in its early stages” [5]. Such species are more common for the early transition metals [6,7], though this is slowly changing [8,9,10,11,12].

Our group has been investigating the syntheses and the catalytic potential of nickel complexes with N-heterocyclic carbene (NHC) ligands over the past two decades and we have prepared and extensively investigated nickel NHC complexes of the type [Ni(Cp)(NHC)(X)] (Cp = η^5^-C_5_H_5_ or η^5^-C_5_Me_5_, X = Cl, Br, I) [13,14]. Most complexes are easy to prepare, as treatment of nickelocene with the appropriate imidazolium salt in a solvent, under reflux or via microwave synthesis, leads directly to [Ni(Cp)(NHC)(X)] species in satisfactory to excellent yields [13,14,15]. These complexes are catalytically active for a wide range of reactions that have been investigated by us and others, including Suzuki–Miyaura coupling reactions [16,17,18,19,20,21,22], α-ketone arylation [23,24,25], hydrosilylation [14,26,27,28] and hydroboration [29,30,31,32]. We were interested to see if [Ni(Cp)(NHC)(F)] complexes were accessible by using the lesser-known imidazolium fluorides, in an extension of the just discussed method of synthesis.

Nickel NHC species with fluoride ligands may be implicated in cross coupling reactions between aryl Grignard reagents and aryl halides, as NiF_2_ carries out the cross coupling in the presence of imidazolium chloride or tetrafluoroborate salts [33]. In general, however, there are not many isolated nickel NHC complexes with fluoride ligands [9,10,34], so this remains an area that has not been thoroughly investigated.

Imidazolium salts of Cl^−^, Br^−^ and I^−^ are well known and are readily accessible via many synthetic routes. One involves direct alkylation of imidazole using the appropriate alkyl halides in the presence of a base in a classic nucleophilic substitution reaction. For bulkier N-substituted imidazolium salts, an alternative two-step process is used, starting from glyoxal and a primary amine, followed by ring closure of the di-imine produced with formaldehyde and a Lewis acid source. This method often leads to imidazolium chlorides, and both methods have been well investigated [35,36,37]. The Cl^−^ can be replaced with Br^−^ or I^−^ anions by adding KBr and KI [38]. Yields are good to excellent and do not require expensive reagents or anerobic synthetic conditions. However, these synthetic methods do not work for the synthesis of imidazolium fluorides, presumably because of the significant strength of the C–F bonds in the alkyl fluoride.

The first synthetic report of a *N*,*N*′-*bis-*alkyl-imidazolium fluoride appeared in 2012. It involved halide exchange between a *N*,*N*′-*bis-*alkyl imidazolium iodide and silver fluoride [39]. The reaction is driven by the formation of insoluble AgI and was only reported for R = Me and R’ = Me, Et or Pr. Incidentally, similar reactions have been used to prepare metal-fluoro complexes; for example, Pilon and Grushin reported the synthesis of a series of [Pd(PPh_3_)_2_(R)F] complexes that were synthesized this way [40].

Other reports of imidazolium fluorides have since appeared in the literature. While these were sometimes prepared for their properties and use as ionic liquids, over the last ten years, imidazolium fluorides have found increasing applications in organic chemistry towards the nucleophilic fluorination of various substrates [41,42]. Indeed, it is noted, in an article that briefly reviews the field and applies imidazolium fluorides towards nucleophilic fluorination [43], that “the reagent PhenoFluor [44] and its air-stable successor AlkylFluor [45] … seem to be the reagents of choice for deoxyfluorination of phenols and aliphatic alcohols, respectively”. Nevertheless, only a handful of single-crystal X-ray diffraction studies of such fluorides have been reported [39,46]. In this work, we describe the isolation and characterization of three imidazolium fluorides, the structural determination of two of them, and their subsequent reactions with nickelocene, which in fact do not appear to yield [Ni(Cp)(NHC)(F)] species.

## 2. Results and Discussion

### 2.1. Synthesis of the Imidazolium Fluoride Salts ***1a***–***1c***

**Scheme 1:**
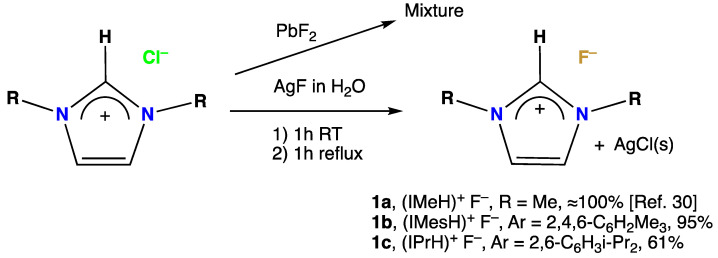
Synthesis of imidazolium fluorides **1a**–**1c** based on Xiao’s method [39].

The salt *N*,*N*′-*bis*-methylimidazolium fluoride, [(IMeH)^+^ F^−^] **1a**, which, incidentally, has been the subject of a theoretical study on its stability [47], was prepared following the procedure reported by Xiao [39] and was obtained in good yield. We attempted to prepare other imidazolium fluorides by an adaptation of this method, using the exchange reaction with PbF_2_ rather than with AgF. The lead salt is not light sensitive and is significantly cheaper. However, this did not work well, as the halide exchange was not quantitative, so we resorted to AgF as the source of fluoride in the exchange reactions for the syntheses of two other imidazolium, fluorides, [(IMesH)^+^ F^−^], **1b**, and [(IPrH)^+^ F^−^], **1c** (IMesH = *N*,*N*′-*bis*-(2,4,6-trimethylphenyl)imidazolium); IPrH = *N*,*N*′-*bis*-(2,6-diisopropylphenyl)imida-zolium). **1c** was prepared by reacting the free carbene *N*,*N*′-*bis*-(2,6-diisopropyl-pheny)imidazol-2-ylidene with KHF_2_ or HF [48], but we preferred avoiding possible HF contact. Our reactions were conducted in glassware wrapped in aluminum foil to minimize light exposure to the light-sensitive silver compounds. The syntheses of these aryl imidazolium fluorides were not clean, as colloidal AgCl tended to form when [(IMesH)^+^ Cl^−^] was treated with AgF. However, careful control of reaction conditions, including the slow addition of a solution of AgF led to relatively pure [(IMesH)^+^ F^−^]. The other salts were similarly prepared. These reactions are outlined in Scheme 1.

The cations in **1b** and **1c** are the same as in the chlorides [(IMesH)^+^ Cl^−^] and [(IPrH)^+^ Cl^−^], but there are significant spectral differences between the F^−^ and Cl^−^ salts due to H- bonding of the C(1)–*H* proton to the fluoride in the fluorides. Indeed, the C(1)–*H* proton, usually observed at *δ* = 9–11 ppm in other imidazolium halides, is usually not seen in ^1^H NMR spectra of **1b** and **1c** though occasionally a very weak broad peak is observed in the 11.5 ppm range. This phenomenon has previously been reported for **1c** [46]. Significant signal broadening could arise from H ^…^ F hydrogen bonding or through H–D exchange due to the presence of traces of water in solution, which is perhaps more likely.

The absence of this signal could conceivably arise from the formation of silver NHC complexes of stoichiometry F–Ag–NHC, but this was refuted by the ^19^F NMR spectra of both species, which exhibit peaks at −131.0 and 131.5 ppm for **1b** and **1c**, not far from the signal at −120.3 ppm observed for free fluoride ions. In addition, there is no evidence of ^107^Ag–F or ^109^Ag–F coupling, and the C–H–N microanalysis negates the presence of silver in these species.

The ^13^C NMR of the imidazolium fluoride salts does not exhibit a signal for the *C(1)*–H carbon atom, possibly due to its coupling to the two flanking quadrupolar nitrogen atoms. All other carbon atoms show their ^13^C NMR peaks in their expected chemical shift range, and there is no evidence of any signals coupling to silver. Furthermore, IR spectra of **1b** and **1c** exhibit strong *ν*(H ^…^ F) peaks at 3640 and 3650 cm*^−^*^1^ respectively.

### 2.2. X-ray Diffraction Studies on the Imidazolium Fluoride Salts ***1b*** and ***1c***

**Figure 1:**
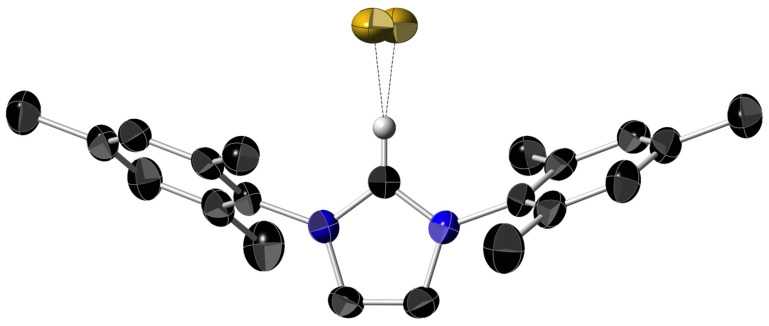
Structure of **1b, [(IMesH)^+^ F^−^]**. **C**, **N** and **F** atoms are shown as **black**, **blue** and **yellow** ellipsoids at the 50% probability level; both positions of the symmetrically disordered **F^−^** ions are shown. Only the imidazolium H-atom is shown as a sphere of arbitrary radius, for clarity, showing its close interactions with the **F^−^** ions (H ^…^ **F** = 1.85 Å).

**Figure 2:**
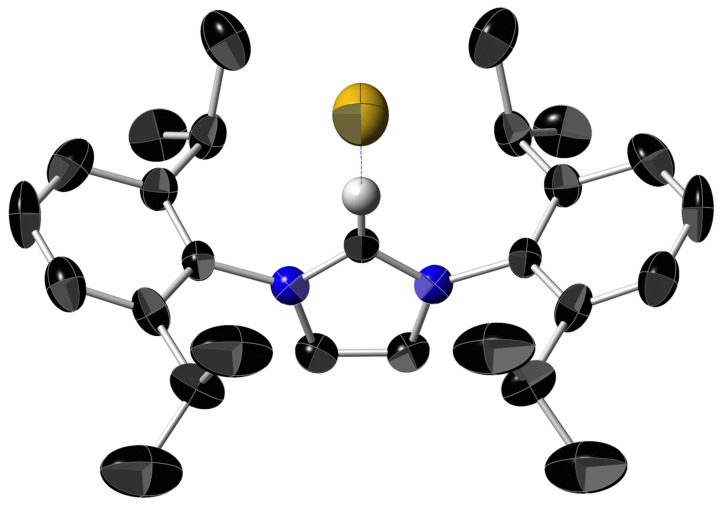
Structure of the salt [**(IPrH)^+^ F^−^**], **1c**. Ellipsoids are shown at the 50% probability level. **C**, **N** and **F** atoms are color coded as shown. Only the imidazolium **H(2)** atom is shown, with a short H ^…^ **F** interaction of 1.86 Å, for clarity.

The H-bonding was confirmed by X-ray diffraction studies on single crystals of **1b** and **1c**, obtained from concentrated CH_2_Cl_2_ solutions. Their structures are shown in Figure 1 and Figure 2. In both cases, there are disordered solvent molecules (H_2_O in **1b**, CH_2_Cl_2_ molecules in **1c**). The structure of **1c** has been previously reported [46], but with acetonitrile in the lattice (as opposed to CH_2_Cl_2_ in ours), and the previously reported cell parameters are (unsurprisingly) different from those we found. Neither structure is of high quality; there is significant disorder in both, that could not be properly modeled. Nevertheless, we believe that they are useful and should appear in the literature, as very few imidazolium fluoride structures are known. Key data for both salts are collected in Table 1 with relevant data for **1a** [39] included as well. Figure 1 and Figure 2 show the molecular structures of the salts. In **1b,** the fluoride atom is disordered symmetrically about two positions.

**Figure 3:**
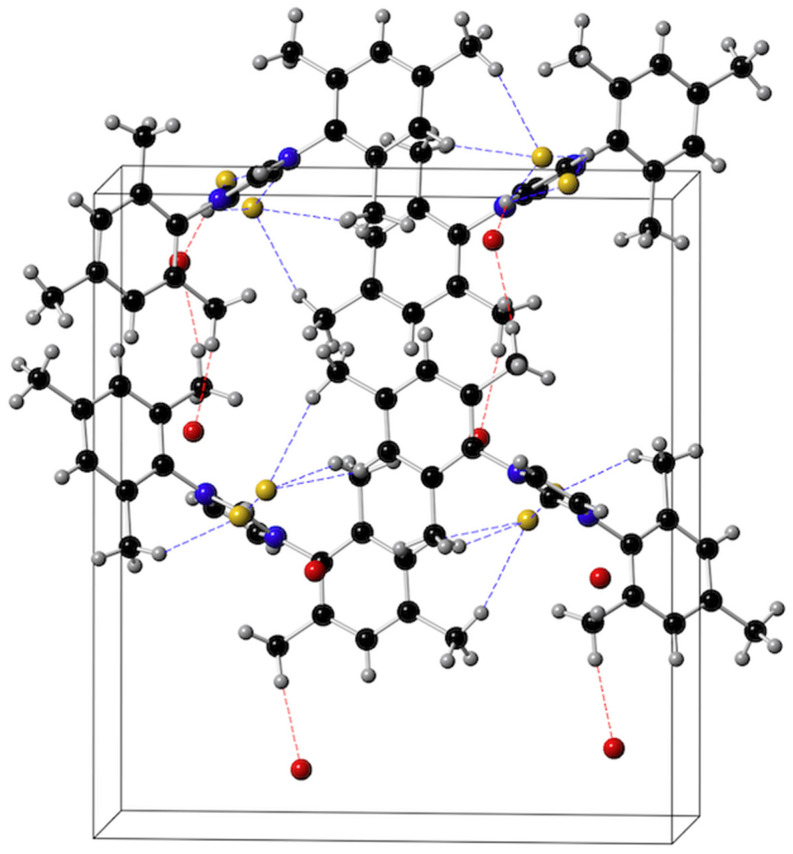
H**^…^**F and H**^…^**O interactions < 3.0 Å in the crystal of **1b**, viewed approximately down the *y*-axis of the unit cell. **C**, **N**, **O** and **F** atoms are color coded.as shown. H_imi_**^…^O** = 2.28, 2.38, 2.53 Å; C(2)H_Imid_**^…^ F** = 1.85; H_Me_**^…^ F** = 2.78, 2.71 Å; H_meta_**^…^ F** = 2.70 Å.

A feature of interest in both structures are the short C(1)_Imid_–H ^…^ F distances of 1.85 and 1.84 Å in **1b** and **1c** respectively, indicative of strong hydrogen bonding. The previously reported structure of **1c** [46] showed a shorter corresponding distance of 1.72 Å, but in all cases, these distances have relatively high estimated standard deviations (esds). The structure of **1a** [39] also shows short C(1)_Imid_–H ^…^ F distances of 1.92 and 2.04 Å. There are many other longer-range H-interactions in both **1b** and **1c**; those of **1b** are shown in Figure 3.

In **1b**, the *meta*-H-atom of the mesityl ring, one of the mesityl CH_3_ hydrogen atoms and the two other C–H hydrogen atoms of the imidazolium ring have longer interactions in the 2.59 to 2.78 Å range with F^−^ ions. There are disordered water molecules, and their O-atoms have shorter interactions of 2.38, 2.28 and 2.53 Å with (respectively) hydrogen atoms of two ortho-CH_3_ groups and one of the imidazolium –CH=CH– atoms that probably help stabilize the structure. The short F ^…^ O distances of 2.474 and 2.537 Å suggest that there are OH^…^F interactions as well, but the water molecules’ hydrogen atoms could not be located or modeled.

**Figure 4:**
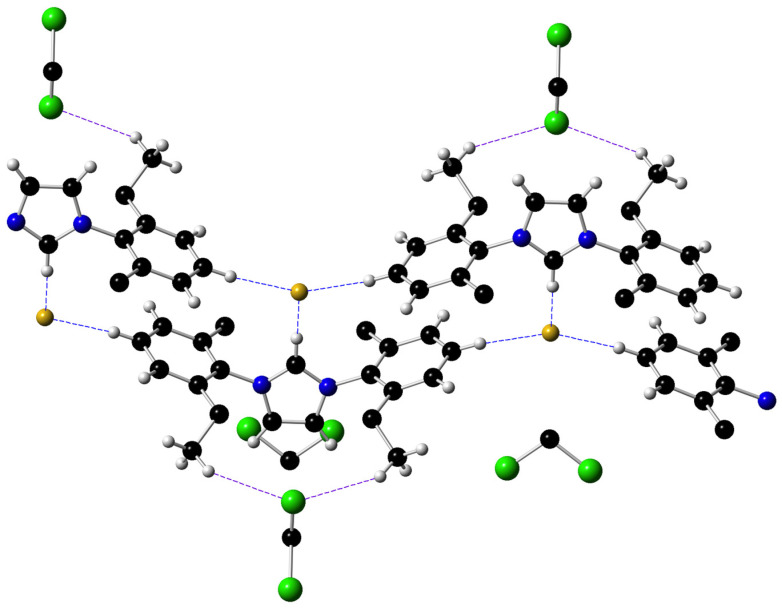
H **^…^ F** and interactions < 3.0 Å in **1c**, viewed down the *z*-axis of the unit cell. **C**, **N**, **Cl** and **F** atoms are color coded as shown. Many **C** and **H** atoms of the i-Pr-group and the CH_2_Cl_2_ H-atoms are hidden, for clarity. H_imi_ **^…^ F** = 1.84 Å; H_para_ **^…^ F** = 2.41 Å.

In compound **1c**, the situation is interesting, as, in addition to the short imidazolium H(2) ^…^ F interaction, mentioned earlier, each para-C–H bond of the two aromatic rings on each imidazolium moiety is linked in a symmetric fashion with the fluoride ion (C–H_para_ ^…^ F = 2.41 Å) as shown in Figure 4. The cations are thus arranged in long chains, effectively linked together via these long H–bonds. The fluoride ion here is in a distorted, but close to trigonal, planar geometry with the C–H_para_ ^…^ F ^…^ C–H_para_ angle being 139°: the two other C–H_para_ ^…^ F ^…^ C(2)–H angles are equivalent and equal to 109° so as to have the fluoride anions surrounded in a very close to trigonal geometry (the sum of the three angles equals 356°). Some of the chlorine atoms of the CH_2_Cl_2_ solvent molecules also have long interactions with some hydrogen atoms: they range from 2.94 to 3.15 Å, though these are probably of marginal significance. There are short F^…^H_2_CCl_2_ interactions of 2.39 Å with one of the dichloromethane hydrogen atoms with the fluoride ion. While occasionally fluoride anions can exhibit short interactions with other halogens [11,49], no F ^…^ Cl_2_CH_2_ short contacts were observed here.

### 2.3. Reactions of Imidazolium Fluoride ***1a*** with Nickelocene and an X-ray Diffraction Study of [Ni(Cp)(IMe)_2_]^+^ F^−^, ***2***

**Scheme 2:**
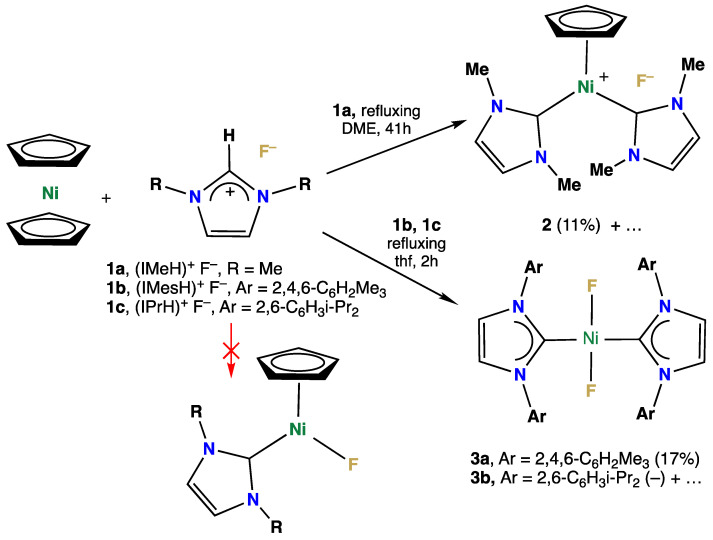
Reactions of the imidazolium fluorides **1a**–**1c** with nickelocene.

The reaction of a wide spectrum of imidazolium halide salts (halide = Cl^−^, Br^−^ or I^−^) with nickelocene has been shown by us and others to lead to the half-sandwich (or two-legged piano-stool) molecules of formula [Ni(Cp)(NHC)(X)], and many of these well-known species have been structurally characterized by us [13,14,16,17] and others [50,51,52,53,54,55]. However, no fluoro-complexes of formula [Ni(Cp)(NHC)(F)] have been reported, and we were interested in exploring the reaction of nickelocene with imidazolium fluorides **1a**–**1c** to try to prepare such a species (Scheme 2).

The reaction was first attempted with **1a**. A relatively rapid color change was quickly observed, with the deep forest green of the nickelocene solution giving way to a cherry red, then purple color within 10 min. More slowly, a dark, almost black, color developed during the total 41 h reaction time. This contrasts with the reaction of the *N*,*N*′-*bis-*methylimidazolium iodide with nickelocene, which is much slower.

After workup (see experimental section), red crystals of **2** were obtained and they exhibited no *ν*(H ^…^ F) in their IR spectrum. ^1^H NMR data indicated the presence of a Cp and two NHC ligands and suggested that **2** was probably the bis-NHC complex [Ni(Cp)(IMe)_2_]^+^ F^−^. The ^13^C NMR spectrum corroborated the ^1^H NMR data and, in addition, exhibited a signal for the carbene carbon atoms at characteristic downfield peak at 162.6 ppm, in line with the signals observed for NHC carbene carbon atoms in other similar *bis*-NHC complexes of nickel, which we have previously observed in the 158–168 ppm range [56]. The ^19^F NMR showed a single peak at −162 ppm, not very close to the signal for free F^−^ in chloroform (which appears at −120 ppm). The F^−^ anion is thus likely to be closely associated with the complex in solution, as some sort of ion pair.

**Figure 5:**
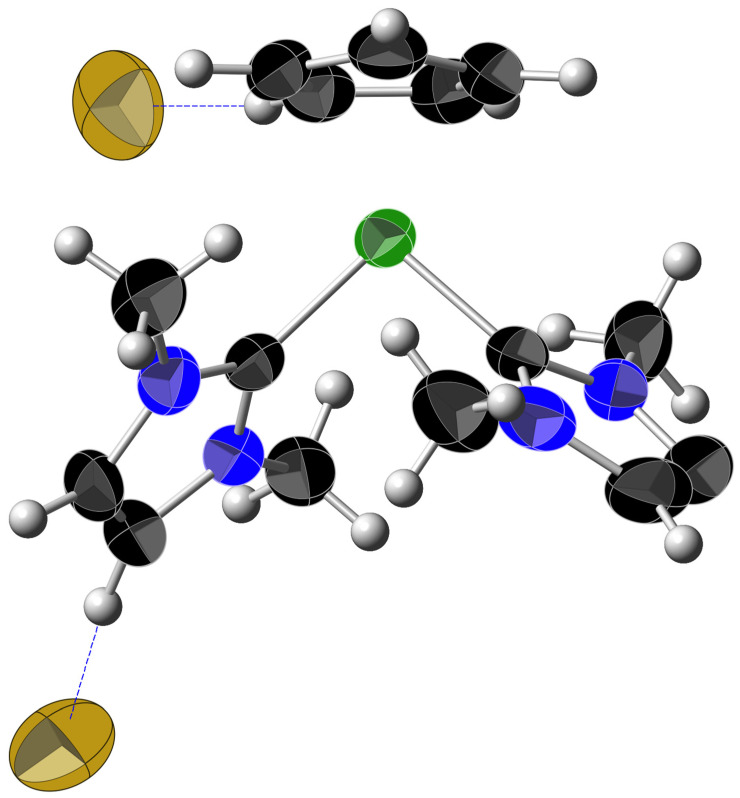
Structure of **2**, [Ni(Cp)(IMe)_2_]^+^ F^−^. Ellipsoids are shown at the 50% probability level and the **C**, **N**, **Ni** and **F** atoms are colored as shown. Two relatively short H **^…^ F** interactions are shown (H_Imid_ **^…^ F** = 2.28 Å, H_Cp_ **^…^ F** = 2.41.

The structure of [**Ni(Cp)(IMe)_2_]^+^ F^−^**, **2** (Figure 5) was confirmed by single-crystal X-ray diffraction on a crystal grown by slow diffusion of Et_2_O into a CH_2_Cl_2_ solution of **2**. Key structural parameters are collected in Table 2. The cation forms a 2-legged piano stool, whose structure is similar to those of other [NiCp(NHC)X]^+^ (X = Cl, Br, I, NHC’) species described by us [13,14,16,17,56] and others [15,50,51,52,53,54,55]. The C_carb1_–Ni–C_carb2_ angle is 94.5°, which is in the range of what is seen in other complexes of this geometry (96.9° is observed for this angle in [Ni(Cp)(IMes)(IMe)]^+^) [56]. As is also commonly observed in [Ni(Cp)(NHC)_2_]^+^ salts [15,56], if one takes the centroid of the Cp ligand and the two carbene carbon atoms, the nickel is in the center of a very close to trigonal planar geometry [the sum of the three angles subtended by the nickel atom to these three centers is 359.9°].

**Figure 6:**
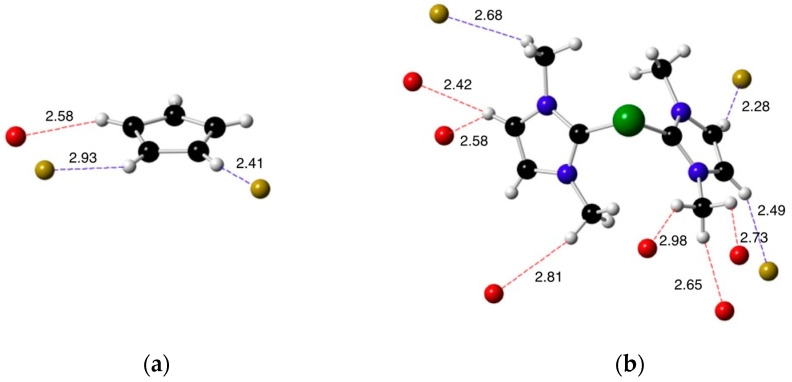
Hydrogen short contacts and H-bonds (in Å) for (**a**) the *η*^5^-C_5_H_5_ and (**b**) the Ni(IMe)_2_ portions of complex **2** with the **F^−^** anion and the **O** atom of H_2_O molecules present in the lattice. **C**, **N**, **Ni**, **F** and **O** atoms are colored as shown.

**Figure 7:**
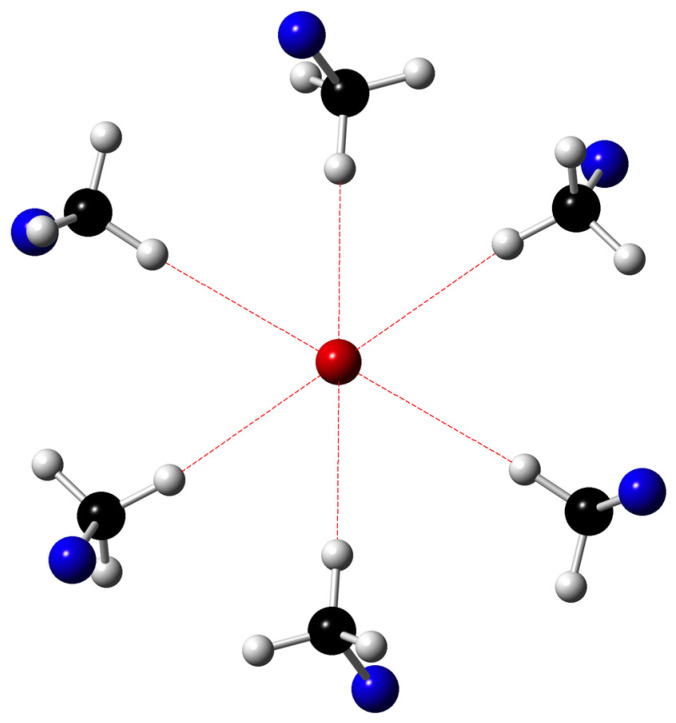
View down the crystallographically imposed 3-fold rotational *z*-axis in **2** that shows the pseudo-octahedral geometry (with a significant rhombic distortion) of an oxygen atom of water, that interacts weakly with 6 methyl group hydrogen atoms of 6 different IMe groups. All H ^…^ **O** = 2.81 Å; H^…^**O**^…^H angles are 74, 105 or 180°. **C**, **N**, **O** atoms are colored as shown with H atoms shown as small grey spheres.

There are many significant supramolecular H-interactions in the structure that lead to a three-dimensional network in the crystal. While such phenomena were also observed in the structures of the two imidazolium fluorides described earlier in this manuscript, the hydrogen interactions are much more extensive here. The salt crystallizes in the high symmetry trigonal space-group *R*_3_, and an imposed three-fold rotational axis relates each cation to the other two that are ordered around it, via this axis. There are also three disordered water molecules incorporated in the structure. Short H ^…^ F distances of 2.28 and 2.49 Å are seen for a fluoride ion that is effectively bridging two C(H)=C(H) hydrogen atoms from two different carbene ligands on two different cations, via this H-bond interaction. One of the two C(H)=C(H) atoms of the other carbene ligand that is not involved in a H ^…^ F interaction instead shows two short interactions of 2.45 and 2.58 Å with two oxygen atoms of the water molecules present. There are also short interactions between some of the relatively acidic Cp hydrogen atoms with the fluoride ions (2.41 Å) and with the oxygen atoms of the water (2.58 Å). Even some of the sp^3^-carbon bonded hydrogen atoms of the dimethylated imidazolyidene methyl groups are involved with these electronegative elements, and H_Me_ ^…^ F distances of 2.68 Å are seen for H^…^F distances and 2.72, 2.97 and 2.65 Å for H^…^O distances. A water molecule’s hydrogen atom is also strongly interacting with the fluoride ion (F^…^H–O–H = 1.72Å). These interactions are collected in Figure 6. Finally, and remarkably, six hydrogen atoms, from different N–CH_3_ groups, are pseudo-octahedrally coordinated to a (water) oxygen atom with H ^…^ O distances all equivalent to 2.81 Å in a hexagonal channel (Figure 7).

The base-assisted deprotonation of the imidazolium salt to the free carbene was initially hypothesized to be the first step in the synthesis of **2**. We have previously reported the protonation of a η^5^-C_5_H_5_ group with HCl, and the subsequent loss of free cyclopentadiene in an unusual reaction of an 18-electron nickel complex [57], so such a reaction mechanism is not unprecedented. In addition, nickelocene is formally a 20-electron complex which makes the Cp ligand much more reactive and labile. As 60% yields of **2** were obtained using a NiCp_2_: **1a** ratio of 1:2, Cp^−^ is unlikely to be the base, as a maximum yield of 50% would be expected. Furthermore, with a NiCp_2_: **1a** ratio of 1:4, 96% yield could be obtained. The HF produced could protonate one of the nickel cyclopentadienyl ligands to give free cyclopentadiene, and the process would then be repeated for the coordination of the second carbene.

### 2.4. Reactions of the Imidazolium Fluorides ***1b*** and ***1c*** with Nickelocene and an X-ray Diffraction Study of trans-[NiF_2_(IMes)_2_], ***3a***

Cationic nickel complexes of stoichiometry [Ni(Cp)(NHC_1_)(NHC_2_)]^+^ were reported by us and by others as previously mentioned [15,56]. The two NHC ligands can be different from each other, but they all share one characteristic feature: the two NHC ligands are relatively small. Indeed, our failed attempts to prepare the complex [Ni(Cp)(IMes)_2_]^+^ led us to discover an interesting base-promoted C–H activation of acetonitrile [58].

The reaction of **1b**, [(IMesH]^+^ F^−^] with nickelocene thus was unlikely to lead to [Ni(Cp)(IMes)_2_]^+^ cations and this, we believed, increased the odds of preparing a complex of formula [Ni(Cp)(NHC)(F)]. The reaction of two equivalents of **1b** with nickelocene was rapid: within 10 min of mixing, the deep green color of nickelocene gave way to a red color. Complex **3a** was isolated as orange-red powder in relatively poor yield (17%) after workup. However, no cyclopentadienyl signals were observed in the ^1^H NMR spectrum. There were resonances consistent with one (or more than one equivalent) IMes carbene ligand that was presumably coordinated to the nickel. The spectrum was not clean and appeared to show at least two products as well as free **1b**; the quantity of the second minor product increased with time. Reactions of **1b** and **1c** with nickelocene are shown in Scheme 2.

Clearly, **3a** is not stable in solution, as decomposition products rapidly formed within minutes. Despite repeated attempts, we were unable to characterize any of these other products. A few crystals of **3a** could be obtained and a ^1^H NMR spectrum of these dissolved crystals was obtained at low temperature

The ^1^H NMR spectrum showed signals that corresponded to the major product in the crude reaction mixture, but it had broad peaks, possibly suggesting a paramagnetic impurity (see Supplementary Materials); the sample degraded at room temperature affording **1b** and uncharacterizable decomposition products. It was not possible to obtain a clean (and non-broadened) spectrum of **3a**, even at low temperatures. The ^13^C NMR spectrum was also messy and no carbene carbon signal could be seen. A ^19^F NMR spectrum exhibited a signal at −157 ppm and a small unidentified peak at −113 ppm. As pointed out by a thorough reviewer, fluorides often react with borosilicate glass with its traces of adsorbed water to give [SiF_5_]^−^ and perhaps [SiF_6_]^2−^ and [BF_4_]^−^ [4]. This could account for the instability of **3a** in solution and the unidentified peak as a decomposition product.

The first reported ^19^F chemical shift of [SiF_5_]^−^ was given as +136.7 ppm [59] and later at +136.0 [60]. Discrepancies in ^19^F NMR chemical shift values have been the subject of a recent publication [61]; furthermore, older publications may have a sign convention problem, so the quoted values may in fact be at negative ppm values. The ^19^F chemical shifts of [SiF_5_(H_2_O)]^−^ and [SiF_6_]^2−^ are reported at −130.5 and −129.5 ppm, respectively [62], and that of [BF_4_]^−^ at −139.8 ppm) [63], so the unidentified peak cannot be attributed to any of them. However, it cannot be ruled out that the instability of the compound results from reactions with the borosilicate glass surface.

**Figure 8:**
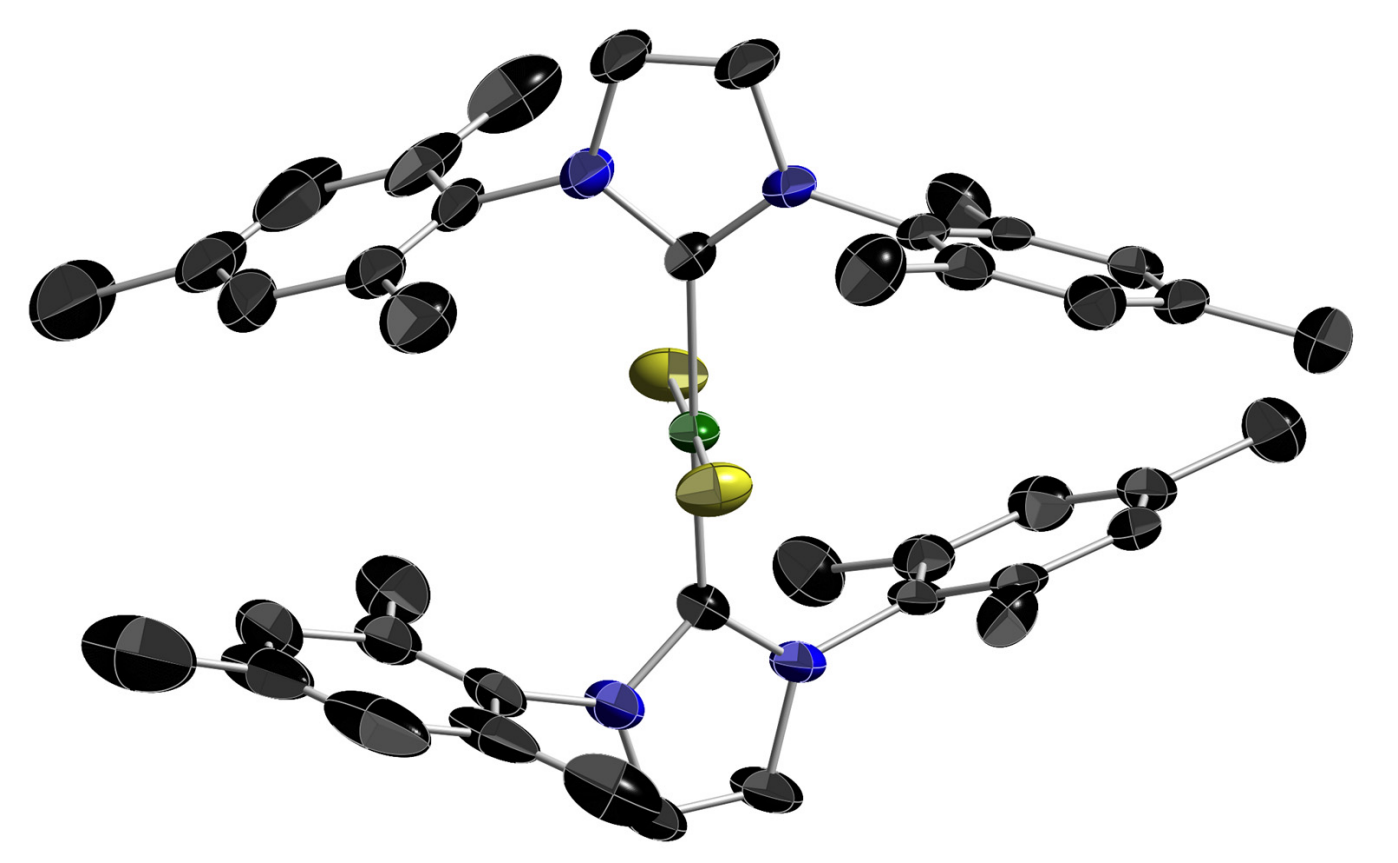
Structure of complex **3a**, *trans*-[NiF_2_(IMes)_2_]. Ellipsoids are shown at the 50% probability level. **C**, **N**, **F** and **Ni** atoms are colored as shown. Hydrogen atoms are not shown for clarity.

A single crystal X-ray diffraction study on an isolated crystal established that **3a** is the square planar Ni(II) complex *trans-*[NiF_2_(IMes)_2_]. Complexes of generic formula [NiX_2_(NHC)_2_] (X = Cl, Br, I) are well known [64], but to our knowledge this is the first example in which X = F. The structure is shown in Figure 8; key structural parameters of **3a** and of a few other square-planar Ni-NHC complexes that contain a single fluoride as a ligand are collected in Table 3.

Only a handful of Ni(NHC) complexes bearing fluoride ligands are known [9,10,25], and to our knowledge, this is the first example of a Ni-NHC complex with two fluoride groups coordinated to nickel. In complex **3a**, the two halves of the molecule are equivalent via the crystallographically imposed symmetry from the high symmetry trigonal space group *P*$\bar{3}$*c1*. The nickel coordination geometry is similar to those of square planar *trans*-[NiX_2_(NHC)_2_] complexes that have been characterized with other halide ligands [65], and to [*trans-*[Ni(NHC)_2_(aryl)(F)] complexes (shown in Table 1) [9,10]. The F–Ni–F angle is 178.3 (18)°, while the F–Ni–C_carb_ angles are 90.79 (13) and 89.23 (13)°. These minor distortions are likely due to the steric pressure exerted by the relatively large IMes ligands. Furthermore, as is seen with all other structures presented in this paper, each fluorine atom has close contacts (six in all) with neighboring methyl hydrogen atoms that range from 2.466 to 2.659 Å (mean distance = 2.553 Å). The Ni–F distance of 1.816 (2) Å is significantly shorter than that observed for other complexes shown in Table 3, probably because of the less significant steric strain exerted by the smaller IMes ligand as compared to IPr. The mesitylene ligands on each carbene are not in the plane of the NHC ligand but are twisted at angles of 76 and 84° with respect to it.

**Figure 9:**
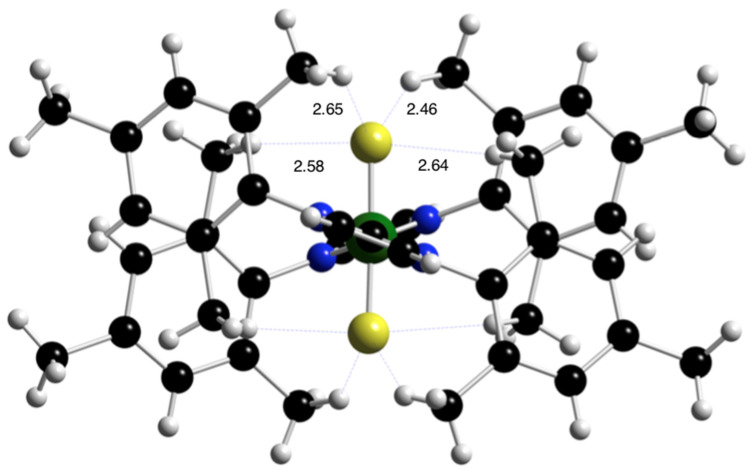
Intramolecular H ^…^ **F** interactions in **3a**, *trans*-[NiF_2_(IMes)_2_]. **C**, **N**, **F** and **Ni** atoms are color coded as displayed. Only H atoms (of arbitrary radius) on the *ortho*-Me groups are depicted, for clarity. *Intramolecular* close contacts are shown (distances in Å). *Intermolecular* H ^…^ **F **interactions of 2.49 (with a *meta-*hydrogen) and with a CH_3_ group hydrogen atom of 2.47 are not shown.

As is seen with all other structures in this paper, the fluorine atoms have close contacts (six in all) with neighboring hydrogen atoms. These distances range from 2.46 to 2.65 Å (mean = 2.58 Å), and four of these (per fluorine) are intramolecular, coming from four different hydrogen atoms from four different *ortho*-methyl hydrogens on each mesityl group. The remaining two are intermolecular, one from an *ortho*-methyl hydrogen atom (at a distance of 2.47 Å) and the other from the *meta*-CH aromatic hydrogen atom (2.49 Å). The intramolecular H-interactions are shown in Figure 9.

Some evidence suggests that the analogous reaction of the imidazolium salt **1c**, [(IPrH)^+^ F^−^], with nickelocene affords the analogous square planar complex **3b**, *trans-*[NiF_2_(IPr)_2_]. However, this species was not fully characterized. Despite repeated attempts, it could not be obtained pure and free of the imidazolium salt **1c**. However, the ^1^H NMR spectrum of the impure product was obtained and is included in the Supplementary Materials together with its IR spectrum. Perhaps the increased steric footprint of the large IPr ligands contributes to this instability in solution.

### 2.5. Catalytic Tests

**Scheme 3:**
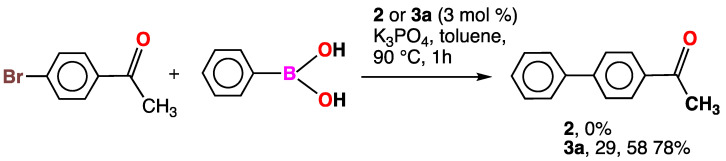
Catalytic tests of **2** and **3a** for Suzuki–Miyaura coupling.

Preliminary catalytic studies were carried out on complexes **2** and **3a**. The coupling reaction between phenylboronic acid and 4-bromoacetophenone was implemented at 90 °C in toluene with the addition of 3 mol% of either **2** or **3a** as the catalyst and the addition of K_3_PO_4_ as a base (Scheme 3). After 1 h, an aliquot of the reaction mixture was taken and analyzed by ^1^H NMR using methods previously described [16,17,18].

Complex **2** did not lead to any coupling product, as only starting organic materials could be observed in the ^1^H NMR spectrum. This is not entirely surprising, as previous work in our labs has shown that [Ni(Cp)(NHC)_2_]^+^ X^−^ complexes have been ineffective in exhibiting catalytic activity towards Suzuki–Miyaura coupling [56]. This is probably due to the high stability of having two strongly *σ*-bonded NHC ligands on the nickel atom (an electronic effect) as well the overall steric protection of the nickel center, which is surrounded by two NHCs and a η^5^-C_5_H_5_ ligand. This prevents the coordination of starting materials and any possible ring-slippage of the η^5^-C_5_H_5_ group to provide a vacant coordination site, or reduction to catalytically active Ni(0) species.

The square planar complex **3a**, *trans*-[NiF_2_(IMes)_2_] was catalytically active, but widely varying yields of coupling products were observed in three catalytic runs with conversion percentages of 29, 58 and 78%. Furthermore, as has already been noted, **3a** decomposes in solution, and color changes from pale orange to light brown were observed in each catalytic run. No further catalytic experiments were performed.

The high chemical stability of **2** was shown by no observable degradation of the complex in its attempted protonation using 0.01 M HCl(aq) or 0.01 M HNO_3_(aq) when stirred at room temperature for 1 h with these acids. In both cases, no color changes were observed and only signals for **2** were observed after the acid exposure. Experimental details of the catalytic studies and on the inertness of 2 towards mineral acids are given in the “Materials and Methods” section below.

## 3. Materials and Methods

Reactions were carried out using standard Schlenk techniques, anaerobically under argon unless otherwise stated. All chemicals were purchased from the Sigma-Aldrich chemical company (Saint Quentin, France) or Fisher (Illkirch, France). Commercial compounds were used as received. Solvents were distilled over sodium/benzophenone (diethyl ether, thf, 1-2-dimethoxyethane), sodium (toluene) or CaH_2_ (pentane, dichloromethane. NMR spectra were recorded at ambient temperature on a Bruker FT 300 or 400 spectrometer (Karlsruhe, Germany) operating at 300.13 or 400 MHz (^1^H), 75.47 or 100.62 MHz (^13^C{^1^H}) and 282.38 MHz for ^19^F NMR spectra in CDCl_3_ unless otherwise stated. Chemical shifts were references to residual solvent peaks for ^1^H and ^13^C NMR spectra and to trifluorotoluene for the ^19^F NMR (set at −63.3 ppm). Chemical shifts (∂) and coupling constants (J) are in ppm and Hz, respectively. IR spectra were collected on a Nicolet 380 FT IR (Thermofisher, Schwerte, Germany) equipped with a diamond SMART ORBIT ATR accessory. Elemental analyses were performed by the Service Analysis Center at the University of Strasbourg. Nickelocene [66] and the salts *N*,*N*′-*bis*-methyl-imidazolium fluoride [39], *N*,*N*′-*bis*-methylimidazolium iodide [17,66] and both *N*,*N*′-*bis*-2,4,6-trimethylphenyl)imidazolium and *N*,*N*′-*bis*-2,6-diisopropylphenyl)imidazolium chlorides were prepared following their literature methods [36,37].

X-ray diffraction data for **1b**, **1c**, **2** and **3a** were all collected on a Bruker APEX II DUO Kappa-CCD diffractometer (Karlsruhe, Germany) equipped with an Oxford Cryosystem liquid N_2_ device, using Mo-Kα radiation (λ = 0.71073 Å) at the University of Strasbourg structural facility. All structures were solved using SHELXS-97 [67,68]. Single crystals of **1b**, **1c** and **2** were obtained by crystallization at 4 °C from dichloromethane (**1b**, **1c**) or by slow diffusion of Et_2_O into a dichloromethane solution of 2. Crystals of **3a** were grown from a thf/pentane solution at room temperature for 12 h and then at 4 °C for 1 day. The crystal–detector distance was 38 mm. The cell parameters were determined (APEX2 software) [69] from reflections taken from three sets of six frames, each at 10 s exposure. The refinement and all further calculations were carried out using SHELXL-2019 [70]. The H-atoms were included in calculated positions and treated as riding atoms using SHELXL default parameters. The non-H atoms were refined anisotropically, using weighted full-matrix least-squares on F^2^. A semi-empirical absorption correction was applied using SADABS in APEX2 [55]; transmission factors: T_min_/T_max_ = 0.8198/0.9265 for **3a**, T_min_/T_max_ = 0.6805/0.7456 for **1b**, T_min_/T_max_ = 0.6383/0.7456 for **1c**, T_min_/T_max_ = 0.6700/0.7456 for **2**. For compounds **2** and **3b**, the SQUEEZE instruction in PLATON [71] was applied. Residual electron density was assigned to one molecule of water present in the crystal for compound **2**.

### 3.1. Syntheses and Spectroscopic Data

All imidazolium fluoride salts were prepared similarly, by an adaptation of Xiao’s method [39]. As the silver salts are photosensitive, glassware was protected with aluminum foil to prevent their photodecomposition.

### 3.2. N,N′-bis-(2,4,6-Trimethylphenyl)imidazolium Fluoride, [IMesH^+^ F^−^], ***1b***

AgF (272 mg, 2.14 mmol) was dissolved in H_2_O (20 mL) and [IMesH^+^ Cl^−^] (733 mg, 2.15 mmol) was added slowly. The mixture was stirred for 1 h and then refluxed for 1 h. The solution was then cooled in an ice-bath, filtered, and the water was removed under reduced pressure. The resulting solid was dried in vacuo overnight to give **1b** (662 mg, 2.04 mmol, 95%) as a cream solid, mpt. 184 °C. ^1^H NMR (CDCl_3_): 11.30 (? C*H*^…^F), 7.57 (2H, im, *H* 4, 5), 6.95 (4H, *meta*-*H*), 2.28 (6*H*, *para*-C*H_3_*), 2.08 (12*H*, *ortho-*C*H*_3_). ^13^C{^1^H}: 141.0 (*ipso-C*_Ar_), 134.2 (*ortho*-*C*_Ar_), 130.9 (*para*-*C*_Ar_), 129.7 (*meta*-*C*_Ar_), 124.3 (im *C4, 5*), 21.1 (*para*-*C*H_3_), 17.4 (*ortho*-*C*H_3_). ^19^F{^1^H} −131.0. IR: 3642 cm**^−^**^1^ **ν**(H^…^F). Anal: calcd. (expt.) for C_21_H_25_N_2_F.2H_2_O: C, 69.97 (69.48); H, 8.11 (7.80); N, 7.77 (7.43).

### 3.3. N,N′-bis-(2,6-Diisopropylphenyl)imidazolium Fluoride, [IPrH^+^ F^−^], ***1c***

AgF (254 mg, 2.00 mmol) was dissolved in H_2_O (20 mL) and [IPrH^+^ Cl^−^] (850 mg, 2.00 mmol) was added slowly. The mixture was stirred for 2 h and then filtered. The filtrate turned cloudy and was filtered a second time after heating. A milky suspension was still obtained, so it was heated for 2 h and then allowed to sit for 2 d and then filtered a third time. The now clear solution was evaporated under vacuum and deposited a cream-white solid 1c (497 mg, 1.21 mmol, 61%), mpt. 170–173°. ^1^H NMR (CDCl_3_): 7.94 (2H, im, *H* 4, 5), 7.53 (t, 2H, *para*-*H*, *^3^J* = 8.0), 7.30 (d, 4*H*, *meta*-*H*, *^3^J* = 8.1), 2.39 (septet, 4*H*, Me_2_C*H*, *^3^J* = 6.9), 1.22 (d, 12H, *Me*, *^3^J* = 6.3), 1.16 (d, 12H, *Me*, *^3^J* = 6.9). ^13^C{^1^H}:145.3 (*ortho-C*_Ar_), 131.9 (*para*-*C*_Ar_), 130.9 (im *C4, 5*), 130.4 (*ipso*-*C*_Ar_) (124.7 (*meta-C*_Ar_), 29.1 (Me_2_*C*H), 24.6 (*C*H_3_), 23.8 (*C*H_3_). ^19^F{^1^H} −131.5. IR: 3651 cm**^−^**^1^ **ν**(H^…^F). Anal: calcd. (expt.) for C_27_H_37_N_2_F.CH_2_Cl_2_: C, 68.14 (72.69); H, 7.97 (8.87); N, 5.68 (6.06).

### 3.4. Synthesis of [Ni(Cp)(IMe)_2_]^+^ F^−^, ***2***

A solution of nickelocene (384 mg, 2.03 mmol) in 1,2-dimethoxyethane (20 mL) was added to excess *N*,*N*′-*bis-*methylimidazolium fluoride (897 mg, 7.72 mmol) and the mixture was refluxed for 41h. The solution changed rapidly from dark green to deep red within 5 min and then darkened to purple and then to black as the rection proceeded. The solvent was then removed under vacuum and the deep red residue was dissolved in a minimum of toluene and filtered through a 3 × 3 cm Celite pad until the filtrate ran clear. The toluene was then evaporated, and the residue extracted with dichloromethane, again filtered through Celite, and the solvent was removed again under vacuum. A mixture of green and red microcrystals was obtained. Recrystallization from a dichloromethane/diethyl–ether mixture deposited red crystals of 2 (66 mg 0.215 mmol, 11% based on nickelocene). Mpt. (dec.) 130 °C. ^1^H NMR: 7.13 (4H, NC*H*), 5.41 (5H, C_5_*H_5_*), 3.94 (12H, *Me*). ^13^C NMR: 162.6 (N*C*N)*,* 124.8 (N*C*H), 91.2 (*C*_5_H_5_), 38.9 (*Me*). ^19^F NMR: −131.50. Anal: calcd. (expt.) for C_15_H_21_N_4_NiF.2H_2_O: C, 48.55 (48.86); H, 6.32 (6.79); N, 15.10 (14.88).

### 3.5. Synthesis of ***3a***, trans-[NiF_2_(IMes)_2_]

A solution of nickelocene (277 mg, 1.47 mmol) in thf (20 mL) was added to **1b**, [(IMesH^+^ F^−^], 920 mg, 3.06 mmol), and the deep green color of the nickelocene solution gave way to a red-brown color within 10 min. The mixture was refluxed for 2 h and the now deep-red reaction mixture was cooled, filtered through a 3 × 3 cm Celite pad, and washed with thf until the filtrate was clear. The solution was then concentrated and placed in a 4° C refrigerator. Small needles of **3a** (178 mg, 0.252 mmol, 17%) formed on standing overnight. Mpt. (dec.) > 200 °C. ^1^H NMR: 6.99 (8H, *meta-*C*H*), 6.64 (d, 4H, im, *H* 4,5), 2.58 (d, 6H, *para*-C*H*), 1.86 (d, 12H, *ortho*-C*H*). ^13^C NMR: 137.4 (*ipso*-*C*_Ar_)*,* 136.7 (*meta*-*C*_Ar_)*,* 136.6 (*para*-*C*_Ar_)*,* (129.0 *ortho*-*C*_Ar_), 121.9 (N*C*H), 21.6, 17.8 (*Me*). ^19^F NMR: −156.7. Anal: calcd. (expt.) for C_42_H_48_N_4_F_2_Ni: C, 71.50 (70.36); H, 6.86 (7.02); N, 7.94 (7.39).

### 3.6. Catalytic Studies on the Reaction of 4-Bromoacetophenone with Phenylboronic Acid

A Schlenk tube was charged with 3.0 mol % of the catalyst (**2**, 10 mg or **3a**, 21.2 mg) and degassed. 4-bromoacetophenone (1.0 mmol), phenylboronic acid (1.3 mmol) and K_3_PO_4_ (2.6 mmol) were added. Toluene (3 mL) was injected, and the tube was sealed with an unpierced septum and the mixture was immediately placed in a pre-heated oil bath at 90 °C. After 1 h, the reaction was quenched by exposing the reaction medium to air and cooled. ^1^H NMR yields were determined by removing a sample of the supernatant solution with a pipette, filtering the extract into an NMR tube and comparing the methyl signal integrations from the cross-coupling product and the 4-bromoacetophenone. We found that 2 gave 0% conversion, while in three separate experiments, **3a** gave 29, 58 and 78% conversion.

### 3.7. Ligand Lability Studies

Complex 2 was mixed with 1 equivalent of KPF_6_ in acetonitrile (10 mL) and 1 equivalent of 0.01 M HNO_3_ (aq) [or 0.01 M HCl (aq) in a separate experiment]. The mixture was stirred at room temperature for 1h. No color changes were observed. The solvent was then removed under reduced pressure and the crude residue was extracted with CDCl_3_, filtered and then a ^1^H NMR spectrum was obtained. In each case, only signals for 2 were observed.

## 4. Conclusions

These results provide more evidence of the extreme tendency of fluoride or fluoro-complexes of metals to engage in hydrogen bonding in the solid state. In all four structures, there is extensive hydrogen bonding in the crystal. This is not entirely surprising, as fluorine is the most electronegative element, but nevertheless the number of such interactions was unexpected. The reactions of the imidazolium fluorides with nickelocene also show that the fluoride salts do not replicate the chemistry that is observed in these reactions of nickelocene with other imidazolium halides (X = Cl, Br, I). We have been unable to prepare complexes of the type [Ni(Cp)(NHC)(F)] even though a plethora of such complexes are known with many different NHC ligands and X = Cl, Br or I [13,14,15,16,17,18,20,21,22,23]. Furthermore, it appears that *trans*-square-planar complexes of the type *trans-*[NiF_2_(NHC)_2_] are unstable in solution, for reasons that are not clear to us but that may be due to “vessel effects”, i.e., reaction with the borosilicate glass and adsorbed water [4].

## Figures and Tables

**Table 1:** Key parameters of **1b**, **1c**. ^a^ From Ref. [39]; average values of two independent molecules are given. ^b^ Angle between the normal to the least square planes of the aromatic C_6_ ring atoms and the imidazolium C_3_N_2_ atoms.

Parameter (Å or °)	1a, [(IMe)^+^ F^−^] ^a^	1b, [(IMesH)^+^ F^−^]	1c, [(IPrH)^+^ F^−^]
C–H_imid_ ^…^ F	1.92 and 2.04	1.85	1.84
C=C_imid_	1.331 (3)	1.341 (6)	1.360 (17)
C(1)_imid_–N	1.318 (3)	1.332 (3)	1.328 (9)
C(2)_imid_–N	1.362 (3)	1.377 (3)	1.355 (11)
^b^ Ar–imid. least sq. planes angle	–	70.2	82.0

**Table 2:** Structural parameters of complex **2**. ^a^ The average of chemically equivalent bonds is given. ^b^ Angle between the normals of the 2 NHC C_3_N_2_ least square planes.

Parameter (Å, Bond-Distances; Angles,°)	2, [Ni(Cp)(IMe)_2_^+^] F^−^.
^a^ Ni–C_carb_	1.892 (6)
^a^ C=C_imid_	1.320 (11)
^a^ C(1)_imid_–N	1.350 (8)
^a^ C(2)_imid_–N and C(2′)_imid_–N	1.388 (9)
^a^ N–C_Me_	1.463 (9)
^a^ Ni–Cp	2.124 (6)
Ni–Cp_centroid_	1.752
C_carb_–Ni–C_carb_	94.5 (2)
C_carb–_Ni–Cp_centroid_	132.3, 133.1
^b^ IMe(1)–IMe(2) angle	94.5

**Table 3:** Key bond lengths (Å) and angles (°) with estimated standard deviations in parenthesis for *trans*-[NiF_2_(IMes)_2_], **3a** and a few other *trans-*square planar Ni(NHC)_2_ complexes with Ni–F bonds. ^1^ F–Ni^−^C(1) angle. **A** = *trans-*[Ni(*N*,*N*′-(*i*-Pr_2_Im)_2_(C_6_F_4_CF_3_-4)(F)] [10]; **B** = *trans-*[Ni(*N*,*N*′-(*i-*Pr_2_Im)_2_(C_6_F_5_)(F) [9]; **C** = *trans*-[Ni(*N*,*N*′-(*i-*Pr_2_Im)_2_(C_6_F_4_SiMe_3_-4)(F)] [9].

Parameter	3a	A	B	C
Ni–C_carb_	1.905 (4)	1.932 (8), 1.911 (8)	1.924 (2), 1.933 (2)	1.911 (6), 1.905 6)
Ni–F	1.816 (2)	1.856 (4)	1.891 (1)	1.856 (3)
F–Ni–F	178.3 (18)	178.6 (3)	175.5 (1) ^1^	177.7 (2) ^1^
F–Ni–C_carb_	89.23 (13), 90.79 (13)	89.1 (2), 88.0 (3)	90.2 (1), 88.6 (1)	89.4 (2), 89.0 (2)
C_carb_–Ni–C_carb_	178.3 (18)	175.0 (3)	-	175.0 (2)

## Data Availability

The original contributions presented in the study are included in the article/Supplementary Materials.

## Supplementary Materials

The following supporting information can be downloaded at: https://www.mdpi.com/article/10.3390/molecules29184493/s1. Spectroscopic and X-ray data, including the CheckCif files for **1b, 1c**, **2** and **3**. Crystallographic data for compounds **1b**, **1c**, **2** and **3a** have been deposited with the Cambridge Crystallographic Data Centre as supplementary publication nos. CCDC 2383952, 2383953, 2383954 and 2383955. Copies of the data can be obtained free of charge from the Director, CCDC, 12 Union Road, Cambridge CB2 1EZ, UK (fax: +44-1223-336-033; e-mail: deposit@ccdc.cam.ac.uk; http://www.ccdc.cam.ac.uk).

## References

[1] Inoue M., Sumii Y., Shibata N. (2020). Contribution of Organofluorine Compounds to Pharmaceuticals. ACS Omega.

[2] Dolbier W.R. (2005). Fluorine chemistry at the millennium. J. Fluorine Chem..

[3] Zhou L. (2021). Recent Advances in C–F Bond Cleavage Enabled by Visible Ligh Photoredox Catalysis. Molecules.

[4] Nielsen M.M., Pedersen C.M. (2022). Vessel effects in organic chemical reactions; a century old, overlooked phenomenon. Chem. Sci..

[5] Grushkin V.V. (2010). The Organometallic Fluorine Chemistry of Palladium and Rhodium: Studies toward Aromatic Fluorination. Acc. Chem. Res..

[6] Murphy E.F., Ramaswarmy M., Roesky H.W. (1997). Organometallic Fluorides: Compounds Containing Carbon-Metal–Fluorine Fragments of d-Block Metals. Chem. Rev..

[7] Roesky H.W. (1999). Playing the Keyboard of Fluorine Chemistry. Inorg. Chem..

[8] Jasim N.A., Perutz R.N., Whitwood A.C., Braun T., Izundu J., Neumann B., Rothfeld S., Stammler H.-G. (2004). Complexes Contrasting Reactivity of Fluoropyridines at Palladium and Platinum:  C−F Oxidative Addition at Palladium, P−C and C−F Activation at Platinum. Organometallics.

[9] Schaub T., Fischer P., Steffen A., Braun T., Radius U., Mix A. (2008). C-F Activation of Fluorinated Arenes using NHC-Stabilized Nickel(0) Complexes: Selectivity and Mechanistic Investigations. J. Am. Chem. Soc..

[10] Schaub T., Backes M., Radius U. (2006). Catalytic C−C Bond Formation Accomplished by Selective C−F Activation of Perfluorinated Arenes. J. Am. Chem. Soc..

[11] Rachor S.G., Müller R., Kaupp M., Braun T. (2023). Hydrogen and Halogen Bonding to Au(I) Fluorido Complexes. Eur. J. Inorg. Chem..

[12] Gauthier R., Nikolaos V., Tzouras N.V., Zhang Z., Bédard S., Saab M., Falivene L., Van Hecke K., Luigi Cavallo L., Nolan S.P. (2022). Gold N-Heterocyclic Carbene Catalysts for the Hydrofluorination of Alkynes Using Hydrofluoric Acid: Reaction Scope, Mechanistic Studies and the Tracking of Elusive Intermediates. Chem. Eur. J..

[13] Ritleng V., Barth C., Brenner E., Milosevic S., Chetcuti M.J. (2008). Synthesis, structure, and solution dynamics of pentamethyl-cyclopentadienyl nickel complexes bearing N-heterocyclic carbene ligands. Organometallics.

[14] Ulm F., Poblador-Bahamonde A.I., Choppin S., Bellemin-Laponnaz S., Chetcuti M.J., Achard T., Ritleng V. (2018). Synthesis, characterization, and catalytic application in aldehyde hydrosilylation of half- sandwich nickel complexes bearing (κ^1^-C)- and hemilabile (κ^2^-C,S)-thioether functionalized NHC ligands. Dalton Trans..

[15] Abernethy C.D., Clyburne J.A.C., Cowley A.H., Jones R.A. (1999). Reactions of Transition-Metal Metallocenes with Stable Carbenes. J. Am. Chem. Soc..

[16] Ritleng V., Oertel A.M., Chetcuti M.J. (2010). Half-sandwich NHC-nickel(II) complexes as pre-catalysts for the fast Suzuki coupling of aryl halides: A comparative study. Dalton Trans..

[17] Oertel A.M., Ritleng V., Chetcuti M.J. (2012). Synthesis and Catalytic Activity in Suzuki Coupling of Nickel Complexes Bearing *n*-Butyl- and Triethoxysilylpropyl-Substituted NHC Ligands: Toward the Heterogenization of Molecular Catalysts. Organometallics.

[18] Aloui L., Abidi R., Chetcuti M.J. (2020). Synthesis and characterization of nickel-N-heterocyclic carbenes linked to a calix[6]arene platform and their applications in Suzuki-Miyaura cross-coupling catalysis. Inorg. Chim. Acta.

[19] Zell T., Fischer P., Schmidt D., Radius U. (2012). C–Br Activation of Aryl Bromides at Ni^0^(NHC)_2_: Stoichiometric Reactions, Catalytic Application in Suzuki–Miyaura Cross-Coupling, and Catalyst Degradation. Organometallics.

[20] Gu S., Du J., Huang J., Guo Y., Yang L., Xu W., Chen W. (2017). Unsymmetrical NCN-pincer mononuclear and dinuclear nickel(II) complexes of N-heterocyclic carbene (NHC): Synthesis, structure and catalysis for Suzuki–Miyaura cross-coupling. Dalton Trans..

[21] Rahman M.M., Zhao Q., Meng G., Szostak R., Szostak M. (2022). [Ni(Np#)(η5-Cp)Cl]: Flexible, Sterically Bulky, Well-Defined, Highly Reactive Complex for Nickel-Catalyzed Cross-Coupling. Organometallics.

[22] Liao C.-Y., Chan K.-T., Chang Y.-C., Chen C.-Y., Tu C.-Y., Hu C.-H., Lee H.M. (2007). Unexpected Solvent-Induced Cis/Trans Isomerization and Catalytic Application of a Bis-bidentate Nickel(II) Complex with N-Heterocyclic Carbene and Amido Functionalities. Organometallics.

[23] Henrion M., Ritleng V., Chetcuti M.J. (2014). From acetone metalation to the catalytic α-arylation of acyclic ketones with NHC–nickel(II) complexes. Chem. Commun..

[24] Cai Y., Ruan L.-X., Rahman A., Shi S.-L. (2021). Fast Enantio- and Chemoselective Arylation of Ketones with Organoboronic Esters Enabled by Nickel/N-Heterocyclic Carbene Catalysis. Ang. Chem. Int. Ed..

[25] Fernandez-Salas J.A., Marelli E., Cordes D.B., Slawin A.M.Z., Nolan S.P. (2015). General and Mild Ni^0^-Catalyzed α-Arylation of Ketones Using Aryl Chlorides. Chem. Eur. J..

[26] Ulm F., Shahane S., Truong-Phuoc L., Romero T., Papaefthimiou V., Chessé M., Chetcuti M.J., Pham-Huu C., Michon C., Ritleng V. (2021). Half-Sandwich Nickel(II) NHC-Picolyl Complexes as Catalysts for the Hydrosilylation of Carbonyl Compounds: Evidence for NHC-Nickel Nanoparticles under Harsh Reaction Conditions. Eur. J. Inorg. Chem..

[27] Chang A.S., Kawamura K.E., Henness H.S., Salpino V.M., Greene J.C., Zakharov L.N., Cook A.K. (2022). (NHC)Ni(0)-Catalyzed Branched-Selective Alkene Hydrosilylation with Secondary and Tertiary Silanes. ACS Catal..

[28] Junquera L.B., Puerta M.C., Valerga P. (2012). R-Allyl Nickel(II) Complexes with Chelating N-Heterocyclic Carbenes: Synthesis, Structural Characterization, and Catalytic Activity. Organometallics.

[29] Ulm F., Djukic J.-P., Chetcuti M.J., Ritleng V. (2020). Hydroboration of alkenes catalyzed by a nickel N-heterocyclic carbene complex: Reaction and mechanistic aspects. Chem. Eur. J..

[30] Afandiyeva M., Kadam A.A., Wu X., Brennessel W.W., Kennedy C.R. (2022). Synthesis, Structure, and Hydroboration Reactivity of Anionic Nickel(0) Complexes Supported by Bidentate NHC-Pyridone Ligands. Organometallics.

[31] Touney E.E., Van Hoveln R., Buttke C.T., Freidberg M.D., Guzei I.A., Schomaker J.M. (2016). Heteroleptic Nickel Complexes for the Markovnikov-Selective Hydroboration of Styrenes. Organometallics.

[32] Lee B.C., Liu C.-F., Lin L.Q.H., Yap K.Z., Song N., Ko C.H.M., Chan P.H., Koh M.J. (2023). N-Heterocyclic carbenes as privileged ligands for nickel-catalysed alkene functionalisation. Chem. Soc. Rev..

[33] Hatakeyama T., Hashimoto S., Ishizuka K., Nakamura M. (2009). Highly Selective Biaryl Cross-Coupling Reactions between Aryl Halides and Aryl Grignard Reagents: A New Catalyst Combination of N-Heterocyclic Carbenes and Iron, Cobalt, and Nickel Fluorides. J. Am. Chem. Soc..

[34] Schaub T., Backes M., Radius U. (2008). Square-Planar (Pentafluorophenyl)nickel(II) Complexes by Derivatization of a C–F Activation Product. Eur. J. Inorg. Chem..

[35] Jahnke M.C., Hahn F.E. (2010). Chemistry of N-heterocyclic Carbene Ligands. Top. Organomet. Chem..

[36] Arduengo A.J., Krafczyk R., Schmutzler R., Craig H.A., Goerlich J.R., Marshall W.J., Unverzagt M. (1999). Imidazolylidenes, imidazolinylidenes and imidazolidines. Tetrahedron.

[37] Hintermann L. (2007). Expedient syntheses of the N-heterocyclic carbene precursor imidazolium salts Ipr·HCl, IMes·HCl and IXy·HCl. Belstein J. Org. Chem..

[38] Oertel A.M., Ritleng V., Chetcuti M.J. (2009). N′-Activation of *N*-Arylimidazoles: Facile Syntheses of *N*-Alkyl-*N′*-arylimidazolium Iodides from Less Expensive Chloro Substrates. Synthesis.

[39] Zhu Z.-Q., Jiang M.-Y., Zheng C.-G., Xiao J.-C. (2012). Efficient synthesis of 1-alkyl-3-methylimidazolium fluorides and possibility of the existence of hydrogen bonding between fluoride anion and C(sp^3^)–H. J. Fluorine Chem..

[40] Pilon M.C., Grushin V. (1998). Synthesis and Characterization of Organopalladium Complexes Containing a Fluoro Ligand. Organometallics.

[41] Bouvet S., Pégot B., Marrot J., Magnier E. (2014). Solvent free nucleophilic introduction of fluorine with [bmim][F]. Tetrahedron Lett..

[42] Bouvet S., Pégot B., Diter P., Marrot J., Magnier E. (2015). Oxidative desulfurization–fluorination reaction promoted by [bdmim][F] for the synthesis of difluorinated methyl ethers. Tetrahedron Lett..

[43] Alič B., Petrovčic J., Jelen J., Tavčar G., Iskra J. (2022). Renewable Reagent for Nucleophilic Fluorination. J. Org. Chem..

[44] Tang P., Wang W., Ritter T. (2011). Deoxyfluorination of Phenols. J. Am. Chem. Soc..

[45] Goldberg N.W., Shen X., Li J., Ritter T. (2016). AlkylFluor: Deoxyfluorination of Alcohols. Org. Lett..

[46] Alič B., Tavčar G. (2016). Reaction of N-heterocyclic carbene (NHC) with different HF sources and ratios—A free fluoride reagent based on imidazolium fluoride. J. Fluorine Chem..

[47] Huang H., Zhong J., Tan X., Guo X., Yuan B., Lin Y., Francisco J.S., Zeng X.G. (2020). New Insights into the Stability of Anhydrous 2H-Imidazolium Fluoride and its High Dissolution Capability toward a Strongly Hydrogen-Bonded Compound. J. Am. Chem. Soc..

[48] Alič B., Tramšek M., Kokalj A., Tavcar G. (2017). Discrete GeF_5_^−^ Anion Structurally Characterized with a Readily Synthesized Imidazolium Based Naked Fluoride Reagent. Inorg. Chem..

[49] Libri S., Jasim N.A., Perutzm R.N., Brammer L. (2008). Metal Fluorides Form Strong Hydrogen Bonds and Halogen Bonds: Measuring Interaction Enthalpies and Entropies in Solution. J. Am. Chem. Soc..

[50] Kelly R.A., Scott N.M., Díez-González S., Stevens E.D., Nolan S.P. (2005). Simple Synthesis of CpNi(NHC)Cl Complexes (Cp = Cyclopentadienyl; NHC = N-Heterocyclic Carbene). Organometallics.

[51] Abernethy C.D., Cowley A.H., Jones R.A. (2000). Reactions of Nickelocene with 1,3-dimesitylimidazolium chloride. J. Organomet. Chem..

[52] Malan F.P., Singleton E., van Rooyen P.H., Conradie J., Landman M. (2017). Synthesis, structure and DFT conformation analysis of CpNiX(NHC) and NiX_2_(NHC)_2_ (X = SPh or Br) complexes. J. Mol. Struct..

[53] Banach L., Gunka P.A., Zachara J., Buchowicz W. (2019). Half-sandwich Ni(II) complexes [Ni(Cp)(X)(NHC)]: From an underestimated discovery to a new chapter in organonickel chemistry. Coord. Chem. Rev..

[54] Bielinski E.A., Dai W., Guard L.M., Hazari N., Takase M.K. (2013). Synthesis, properties, and reactivity of palladium and nickel NHC complexes supported by combinations of allyl, cyclopentadienyl, and indenyl ligands. Organometallics.

[55] Pankov R.O., Tarabrin I.R., Son A.G., Minyaev M.E., Prima D.O., Ananikov V.P. (2024). Synthesis and comparative study of (NHCF)PdCl2Py and (NHCF)Ni(Cp)Cl complexes: Investigation of the electronic properties of NHC ligands and complex characteristics. Dalton Trans..

[56] Oertel A.M., Ritleng V., Burr L., Chetcuti M.J. (2011). Synthesis and Structural Characterization of Half-Sandwich Nickel Complexes Bearing Two Different N-Heterocyclic Carbene Ligands. Organometallics.

[57] Henrion M., Oertel A.M., Ritleng V., Chetcuti M.J. (2013). Facile displacement of η^5^-cyclopentadienyl ligands from half-sandwich alkyl,NHC–nickel complexes: An original route to robust *cis*-C,C-nickel square planar complexes. Chem. Commun..

[58] Oertel A.M., Ritleng V., Chetcuti M.J., Veiros L.F. (2010). C-H Activation of Acetonitrile at Nickel: Ligand Flip and Conversion of N-Bound Acetonitrile into a C-Bound Cyanomethyl Ligand. J. Am. Chem. Soc..

[59] Gibson J.A., Ibbott D.G., Janzen A.F. (1973). Fluorine NMR spectra of sulfur, phosphorus, and silicon fluorides. Hydrolysis and intra-intermolecular mechanism of fluorine exchange. Can. J. Chem..

[60] Klanberg F., Muetterties E.L. (1968). Nuclear Magnetic Resonance Studies on Pentacoordinate Silicon Fluorides. Inorg. Chem..

[61] Rosenau C.P., Jelier B.J., Gossert A.D., Togni A. (2018). Exposing the Origins of Irreproducibility in Fluorine NMR Spectroscopy. Ang. Chem..

[62] Finney W.F., Wilson E., Callender A., Morris M.D., Beck L.W. (2006). Reexamination of Hexafluorosilicate Hydrolysis by 19F NMR and pH Measurement. Environ. Sci. Technol..

[63] Brownstein S. (1978). Complex fluoroanions in solution. VII. Replacement of fluoride by trifluoroacetate. Can. J. Chem..

[64] Zhang D., Zhou S., Li Z., Wang Q., Weng L. (2013). Direct synthesis of *cis*-dihalido-bis(NHC) complex of nickel(II) and catalytic application in olefin addition polymerization: Effect of halogen co-ligands and density functional theory study. Dalton Trans..

[65] Liu Z.-H., Xu Y.-C., Xie L.-Z., Sun H.-M., Shen Q., Zhang Y. (2011). Controlled synthesis of nickel(II) dihalides bearing two different or identical N-heterocyclic carbene ligands and the influence of carbene ligands on their structures and catalysis. Dalton Trans..

[66] Ritleng V., Brenner E., Chetcuti M.J. (2008). Preparation of a N-heterocyclic carbene nickel(II) complex. Synthetic experiments in current organic and organometallic chemistry. J. Chem. Educ..

[67] Sheldrick G.M. (2008). A short history of SHELX. Acta Crystallogr. Sect. A.

[68] Sheldrick G.M. (1990). SHELXS-97 Program for Crystal Structure Determination. Acta Crystallogr..

[69] (2006). M86-E01078 APEX2 User Manual.

[70] Sheldrick G.M. (2015). Crystal structure refinement with SHELXL. Acta Cryst..

[71] Spek A.L. (2003). Single-crystal structure validation with the program PLATON. J. Appl. Cryst..

