# Supplementary material for: Towards Nickel–NHC Fluoro Complexes—Synthesis of Imidazolium Fluorides and Their Reactions with Nickelocene
Source: Molecules. 2024 Sep 21;29(18):4493. doi: 10.3390/molecules29184493 (PMC11434055; doi:10.3390/molecules29184493)
Supplement: Supplementary file 1 [file molecules-29-04493-s001.zip › molecules-3143643-supplementary.pdf]

Supplementary Information

Towards Nickel-NHC Fluoro complexes - Synthesis of Imidazolium Fluorides and their Reactions with Nickelocene

Siobhan S. Wills, Corinne Bailly [2] and Michael J. Chetcuti [3*]

[1] School of Chemistry, University of New South Wales (UNSW), Sydney NSW 2052 AUSTRALIA siobhan.wills@unsw.edu.au
[2] Fédération de Chimie Le Bel UAR2042, BP20296, 1 rue Blaise Pascal, 67008 Strasbourg cedex, FRANCE. c.bailly@unistra.fr
[3] Organometallic Research Group (OMCAT), LIMA CNRS UMR 7042, ECPM, University of Strasbourg, 25 rue Becquerel, 67087 Strasbourg, FRANCE michael.chetcuti@unistra.fr

* Correspondence: michael.chetcuti@unistra.fr Tel.: 33 3 6885 2631

page 1	Table of Contents
page 2 – 8	^{1}H, ^{13}C and ^{19}F NMR spectra of compounds
page 9 – 11	IR spectra of compounds
page 12	X-ray data collection parameters
page 13	Checkcif of **1b**
page 16	Checkcif of **1c**
page 20	Checkcif of **2**
page 23	Checkcif of **3a**

^{1}H NMR of 1b, $(IMesH)^{+}$ $F^{–}$

^{13}C NMR of 1b, $(IMesH)^{+}$ $F^{–}$

^{19}F NMR of 1b, $(IMesH)^{+}$ $F^{–}$

^{1}H NMR of 1c, $(IPrH)^{+}$ $F^{–}$

^{13}C of 1c, $(IPrH)^{+}$ $F^{–}$

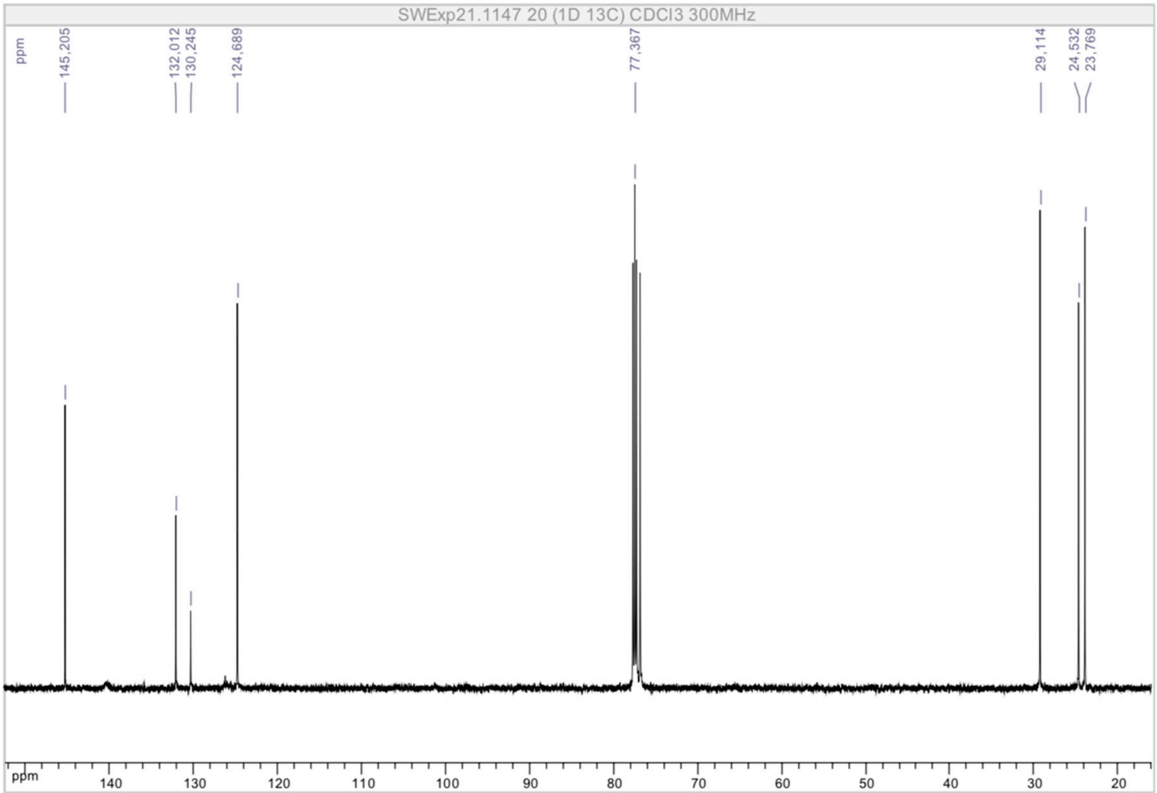

^{19}F of 1c, $(IPrH)^{+}$ $F^{–}$

^{1}H NMR of 2, $[NiCp(IMe)_2]^{+}$ $F^{–}$

^{13}C NMR of 2, $[NiCp(IMe)_2]^{+}$ $F^{–}$

^{1}H NMR of 3a, $[NiF_2(IMes)_2]$ at -50 °C

^{1}H NMR of 3a, $[NiF_2(IMes)_2]$ + decomp. products at 25°C

^{13}C NMR of 3a, $[NiF_2(IMes)_2]$

^{19}F NMR of 3a, $[NiF_2(IMes)_2]$

^{1}H NMR of 3b, $[NiF_2(IPr)_2]$ contaminated with (IPrH)+ F^-

IR Spectrum of 1a, $[IMeH]^+ F^-$

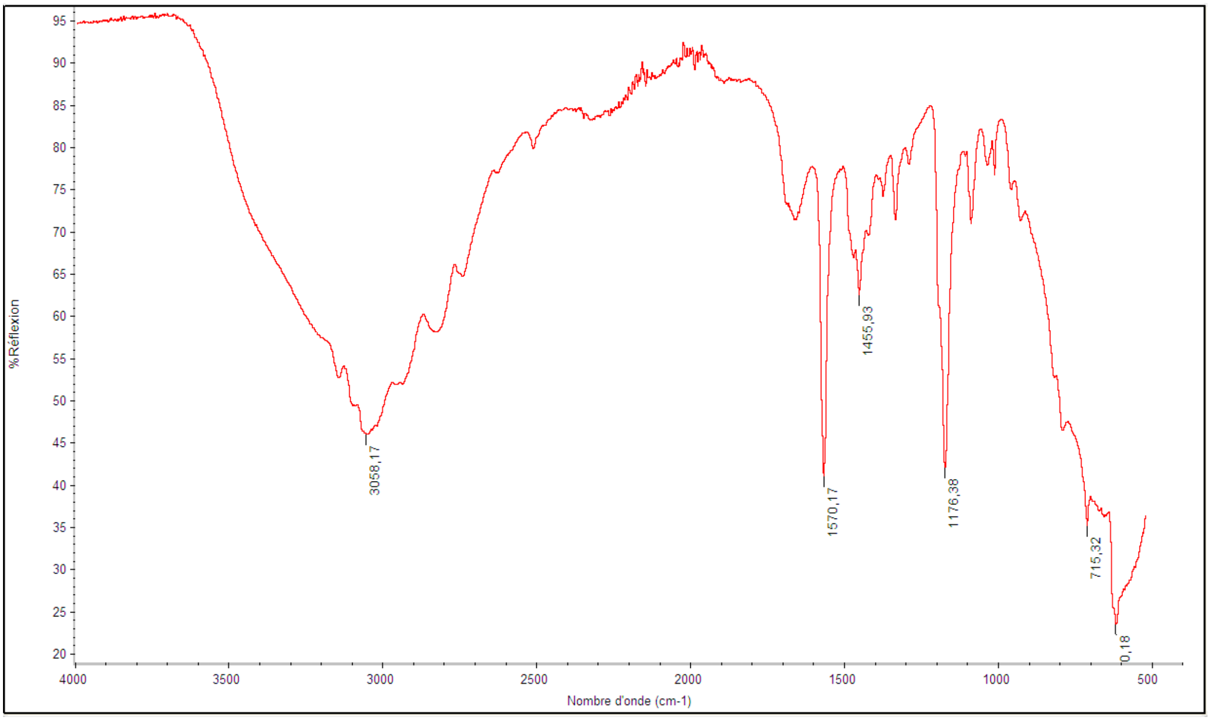

IR Spectrum of 1b, [IMesH]$^{+}$ F$^{–}$

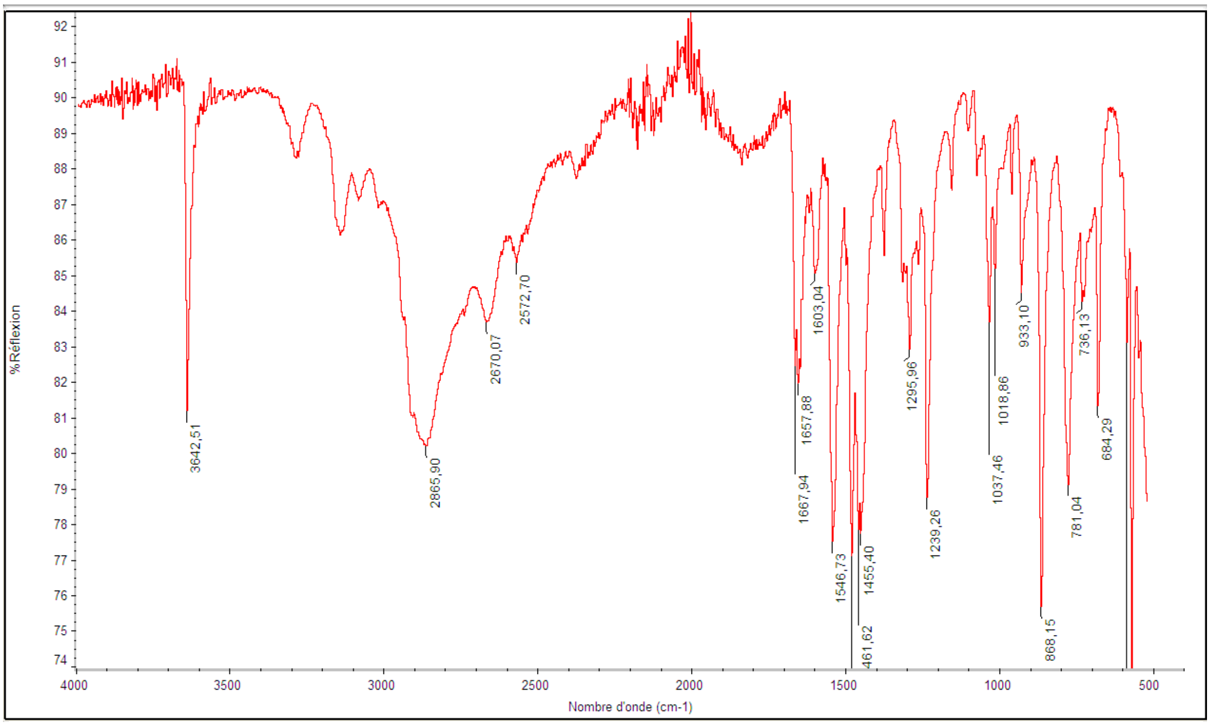

IR Spectrum of 1c, [IPrH]$^{+}$ F$^{–}$

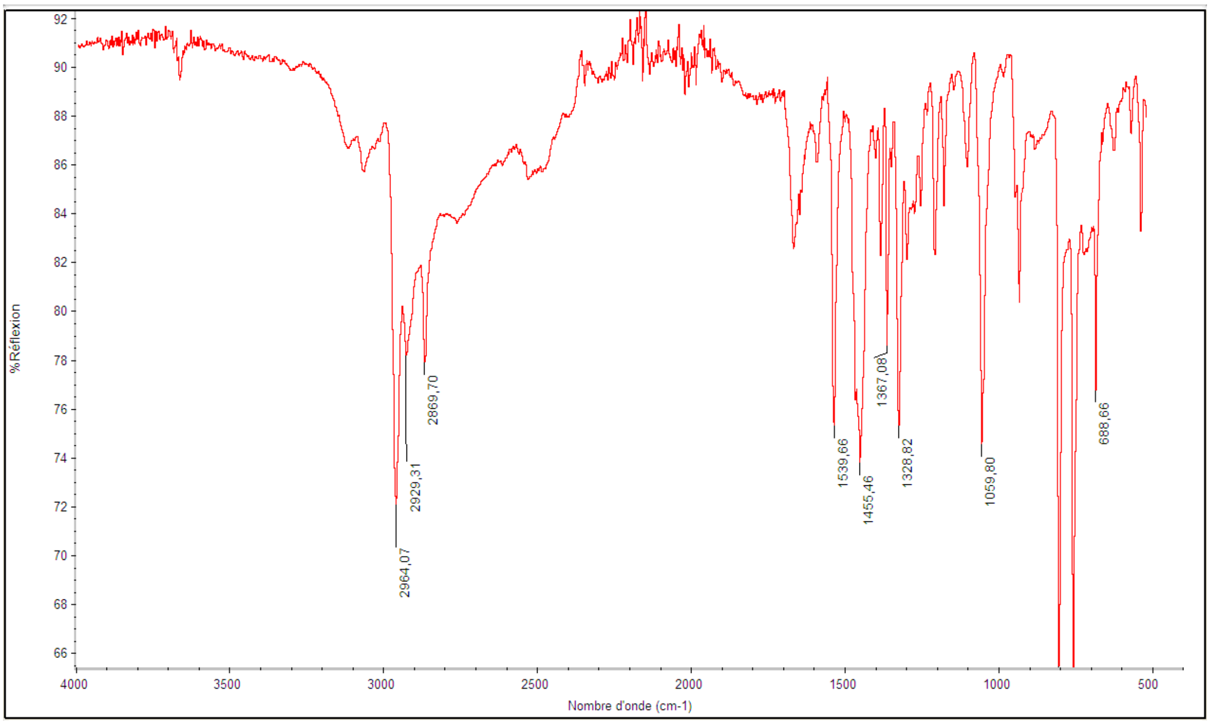

IR Spectrum of 2 $[Ni(\eta^5\text{-}C_5H_5)(IMe)_2]^+$ F^-

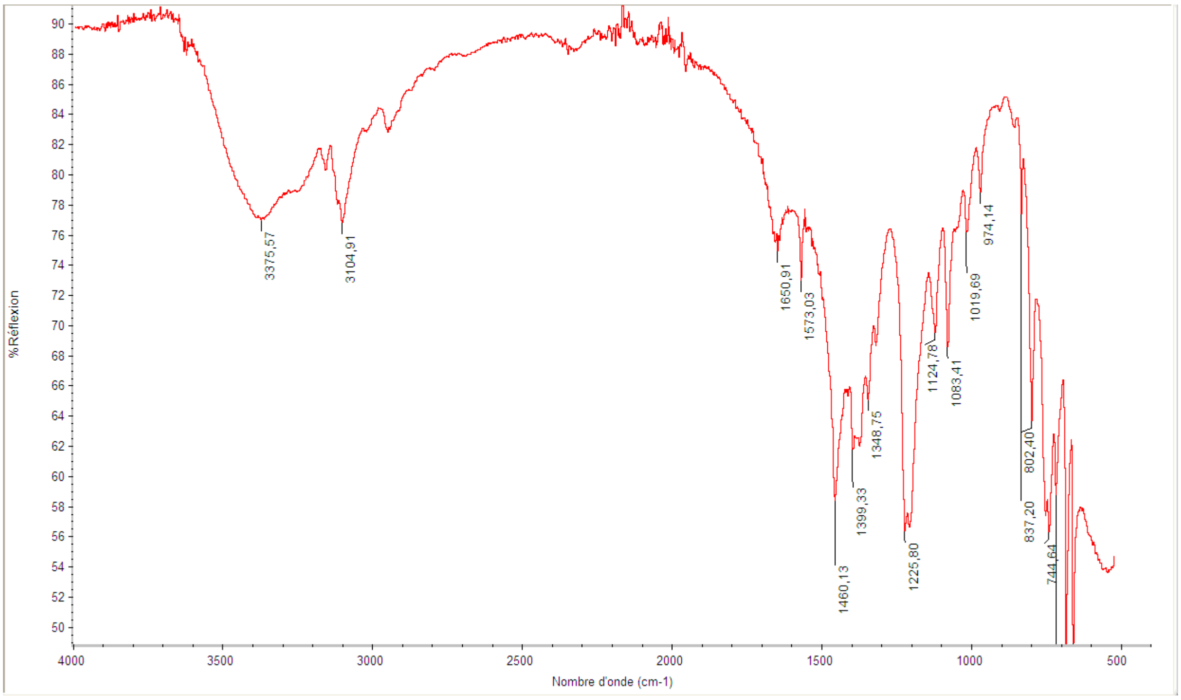

IR Spectrum of 3a, *trans*-$NiF_2(IMes)_2$

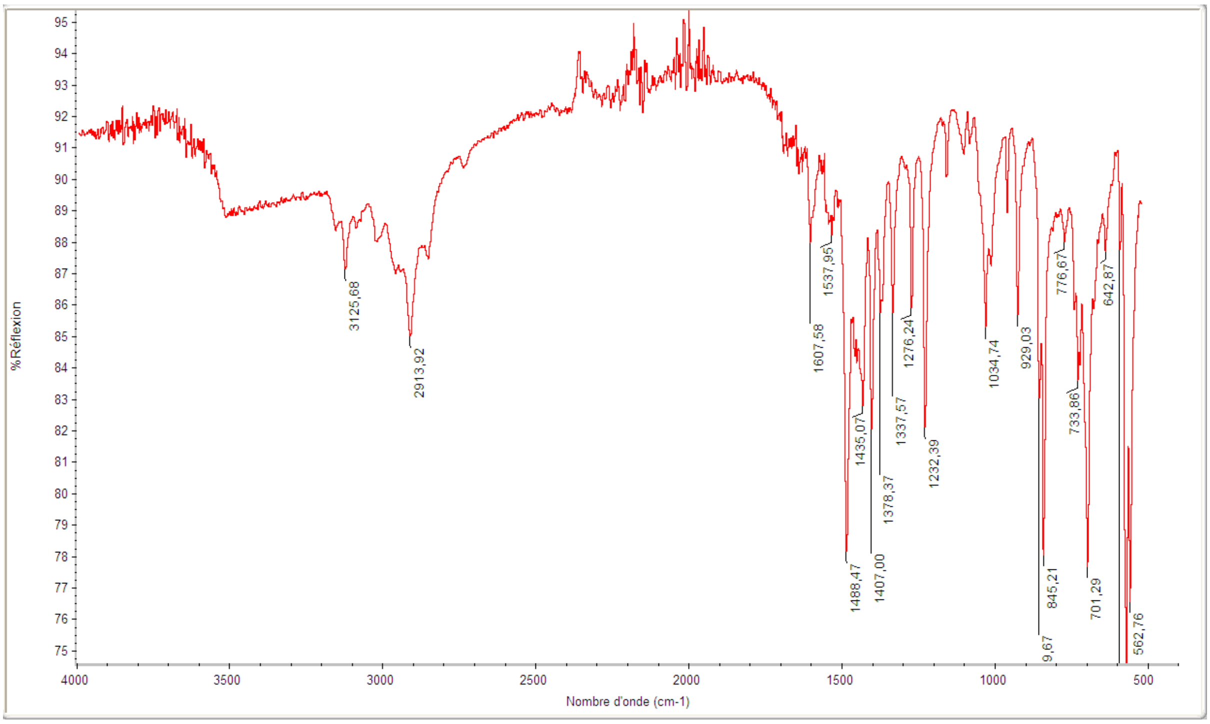

IR Spectrum of 3b, *trans*-$NiF_2(IPr)_2$

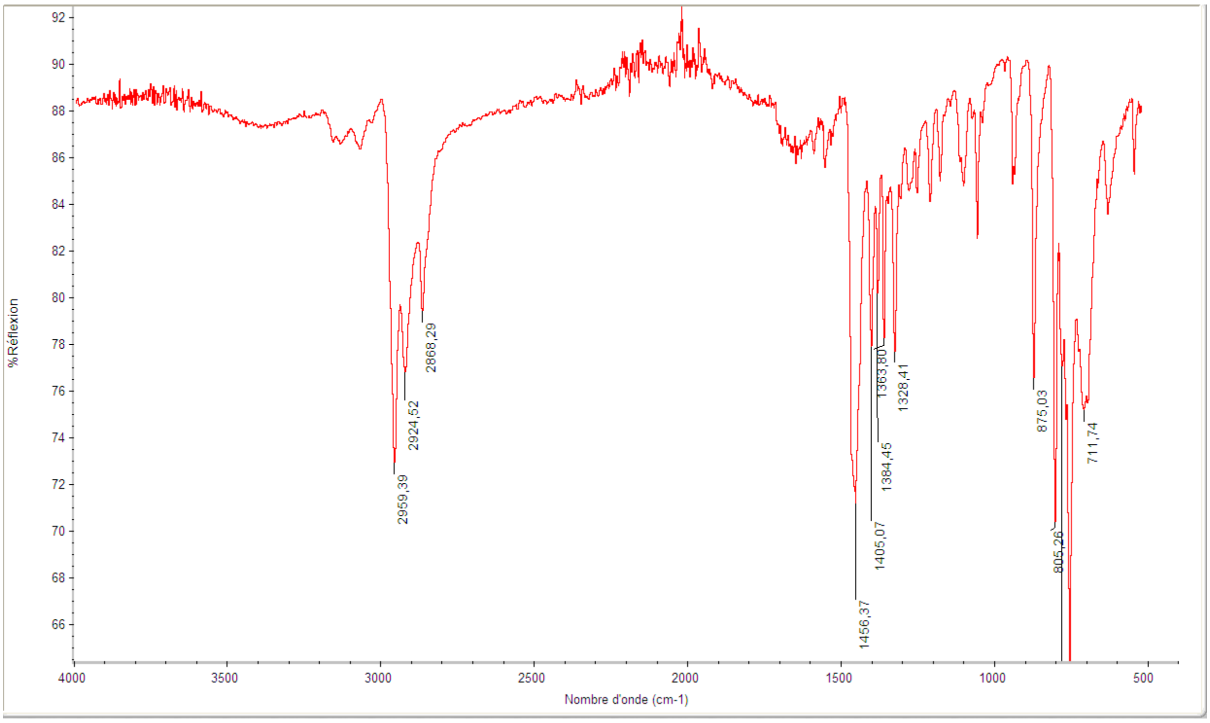

X-ray crystal data for compound 1b, 1c, 2, 3a

Parameter	1b, $(IMesH)^+ F^-$	1c, $(IPrH)^+ F^-$	2 $[NiCp(IMe)_2]^+ F^-$	3, $[NiF_2(IMes)_2]$
Empirical Formula	$C_{21}H_{25}FN_2.2H_2O$	$C_{27}H_{37}FN_2$. $2CH_2Cl_2$	$6(NiC_{15}H_{21}FN_4$. $H_2O)$, 7O	$NiC_{42}H_{48}F_2N_4$
Formula wt. (g mol^{-1}	356.43	578.44	2230.50	705.55
Crystal form. color	Pale yellow block	White block	Red prism	Orange needles
Crystal system	Orthorhombic	Monoclinic	Trigonal	Trigonal
Space group	Pbcn	$P2_1/_m$	$R\bar{3}$	$P\bar{3}c1$
a (Å)<br>*b* (Å)<br>*c* (Å)	16.9849(18)<br>7.8859(7)<br>15.2471(14)	9.3091(12)<br>16.283(2)<br>10.8863(15)	28.2902(17)<br>28.2902(17)<br>12.1774(7)	20.7771(10)<br>20.7777 (10)<br>16.3363(8)
Unit cell angles (°)	90	90, 98.592(3), 90	90, 90, 120	90, 90, 120
Cell volume ($Å^3$)	2042.2(3)	1631.6(4)	8440.3(11)	6107.4(5)
Z	4	2	3	6
X-ray density (g cm^{-3})	1.159	1.177	1.316	1.151
Abs. coefficient (mm^{-1})	0.081	0.388	1.057	0.517
F(000)	760	612	3516	2244
Crystal size (mm)	0.25 x 0.15 x 0.05	0.4 x 0.2 x 0.18	0.5 x 0.2 x 0.2	0.4 x 0.2 x 0.15
Data coll. θ range (°)	2.40, 28.10	2.21, 28.10	2.36, 28.15	1.960, 29.185
Index ranges	$-22 < h < 19$, $-10 < k < 4$, $-20 < l < 20$	$-6 < h < 12$, $-19 < k < 21$, $-13 < l < 14$	$-37 < h < 37$, $-37 < k < 37$, $-7 < l < 16$	$-27 < h < 28$, $-28 < k < 28$, $-22 < l < 22$
Reflections collected	13431	10492	30271	38093
Independent reflections	2485	4100	4566	5444
Completeness to θ (%)	100	99.7	99.9	98.1
Max, min transmission	0.7456, 0.6805	0.7456 0.6383	0.7456, 0.6700	0.9265, 0.8198
Data/restraints/parameters	2485/0/126	4100/15/170	4566/0/214	54440/0/228
Goodness of fit on F^2	0.997	2.545	1.085	1.142
Final R indices [$I > 2\sigma(I)$]	R1 = 0.0718, wR2 = 0.1834	R1 = 0.2690, wR2 = 0.6270	R1 = 0.0776, wR2 = 0.2037	R1 = 0.1018 wR2 = 0.1929
R indices (all data)	R1 = 0.1576 wR2 = 0.2334	R1 = 0.3376 wR2 = 0.6383	R1 = 0.1249 wR2 = 0.2718	R1 = 0.1677, wR2 = 0.2192
Largest diff. peak, hole $Å^3$	0.676, -0.180	6.986, -0.841	1.710, -0.989	1.602, -0.643

X-ray diffraction data were collected at 173(2) K, using Mo-K_α radiation (λ = 0.71073 Å) on a Nonius Kappa CCD diffractometer using graphite-monochromatic Mo K_a radiation (l = 0.71073 Å) at the structural facility of the University of Strasbourg. All structures were refined using SHETLXL-97 [1]. Refinement was via full matrix least squares on F^2. Absorption corrections used the multi-scan method.

[1] Sheldrick, G. M. A short history of SHELX. Acta Crystallogr. Sect. A., 2008, 64, 112-122. DOI: 10.1107/s0108767307043930

CheckCif for **1b**

Checking for embedded fcf data in CIF ...
Found embedded fcf data in CIF. Extracting fcf data from uploaded CIF, please wait **. . . .**

checkCIF/PLATON (basic structural check)

Structure factors have been supplied for datablock(s) **1b**, mcsw120202

IS FOR GUIDANCE ONLY. IF USED AS PART OF A REVIEW PROCEDURE FOR PUBLICATION, IT SHOULD NOT REPLACE THE EXPERTISE OF AN EXPERIENCED CRYSTALLOGRAPHIC REFEREE.

No syntax errors found. CIF dictionary
Please wait while processing .... Interpreting this report
Structure factor report

Datablock: mcsw120202

Bond precision: C-C = 0.0043 A Wavelength=0.71073

Cell:	a=16.9849(18) b=7.8859(7) c=15.2471(14)	
	alpha=90 beta=90 gamma=90	
Temperature:173 K		
Volume	Calculated	Reported
	2042.2(3)	2042.2(3)
Space group	P b c n	P b c n
Hall group	-P 2n 2ab	-P 2n 2ab
Moiety formula	C21 H25 N2, F, 2(O)	C21 H25 N2, F, 2(O) 356.43
Sum formula	C21 H25 F N2 O2	C21 H25 F N2 O2
Mr	356.43	356.43
Dx,g cm-3	1.159	1.159
Z	4	4
Mu (mm-1)	0.081	0.081
F000	760.0	760.0
F000'	760.36	
h,k,lmax	22,10,20	22,10,20
Nref	2499	2485
Tmin,Tmax	0.986,0.996	0.680, 0.746
Tmin'	0.980	

Correction method= # Reported T Limits: Tmin=0.680 Tmax=0.746
AbsCorr = MULTI-SCAN
Data completeness= 0.994 Theta(max)= 28.099
R(reflections)= 0.0718(1207) wR2(reflections)= 0.2334(2485)
S = 0.997 Npar= 126

The following ALERTS were generated. Each ALERT has the format
test-name_ALERT_alert-type_alert-level.
Click on the hyperlinks for more details of the test.

Alert level B

PLAT306_ALERT_2_B Isolated Oxygen Atom (H-atoms Missing ?) ....O1 Check
Disorder precluded their determination.

Alert level C

DIFMX02_ALERT_1_C The maximum difference density is > 0.1*ZMAX*0.75
The relevant atom site should be identified.
PLAT026_ALERT_3_C Ratio Observed / Unique Reflections (too) Low .. 49% Check
PLAT094_ALERT_2_C Ratio of Maximum / Minimum Residual Density .... 3.76 Report
PLAT340_ALERT_3_C Low Bond Precision on C-C Bonds .... 0.0043 Ang.
PLAT906_ALERT_3_C Large K Value in the Analysis of Variance ...... 4.861 Check

Alert level G

PLAT299_ALERT_4_G Atom Site Occupancy Constrained at .... 0.5 Check
F1
PLAT302_ALERT_4_G Anion/Solvent/Minor-Residue Disorder (Resd 2) 100% Note
PLAT304_ALERT_4_G Non-Integer Number of Atoms in ..... (Resd 2) 0.5 Check
PLAT380_ALERT_4_G Incorrectly? Oriented X(sp2)-Methyl Moiety ..... C10 Check

PLAT432_ALERT_2_G Short Inter X...Y Contact F1 ..C1 . 2.78 Ang.
x,y,z = 1_555 Check
PLAT912_ALERT_4_G Missing # of FCF Reflections Above STh/L= 0.600 14 Note
PLAT969_ALERT_5_G The 'Henn et al.' R-Factor-gap value 7.284
Note Predicted wR2: Based on SigI**2 3.20 or SHELX Weight 23.41
PLAT978_ALERT_2_G Number C-C Bonds with Positive Residual Density. 4 Info

0 **ALERT level A** = Most likely a serious problem - resolve or explain
1 **ALERT level B** = A potentially serious problem, consider carefully
5 **ALERT level C** = Check. Ensure it is not caused by an omission or oversight
8 **ALERT level G** = General information/check it is not something unexpected

1 ALERT type 1 CIF construction/syntax error, inconsistent or missing data
4 ALERT type 2 Indicator that the structure model may be wrong or deficient
3 ALERT type 3 Indicator that the structure quality may be low
5 ALERT type 4 Improvement, methodology, query or suggestion
1 ALERT type 5 Informative message, check

It is advisable to attempt to resolve as many as possible of the alerts in all categories. Often the minor alerts point to easily fixed oversights, errors and omissions in your CIF or refinement strategy, so attention to these fine details can be worthwhile. In order to resolve some of the more serious problems it may be necessary to carry out additional measurements or structure refinements. However, the purpose of your study may justify the reported deviations and the more serious of these should normally be commented upon in the discussion or experimental section of a paper or in the "special_details" fields of the CIF. checkCIF was carefully designed to identify outliers and unusual parameters, but every test has its limitations and alerts that are not important in a particular case may appear. Conversely, the absence of alerts does not guarantee there are no aspects of the results needing attention. It is up to the individual to critically assess their own results and, if necessary, seek expert advice.

Publication of your CIF in IUCr journals

A basic structural check has been run on your CIF. These basic checks will be run on all CIFs submitted for publication in IUCr journals (*Acta Crystallographica*, *Journal of Applied Crystallography*, *Journal of Synchrotron Radiation*); however, if you intend to submit to *Acta Crystallographica Section C* or *E* or *IUCrData*, you should make sure that full publication checks are run on the final version of your CIF prior to submission.

Publication of your CIF in other journals
Please refer to the *Notes for Authors* of the relevant journal for any special instructions relating to CIF submission.
PLATON version of 22/08/2024; check.def file version of 21/08/2024

Datablock 1b, mcsw120202 - ellipsoid plot

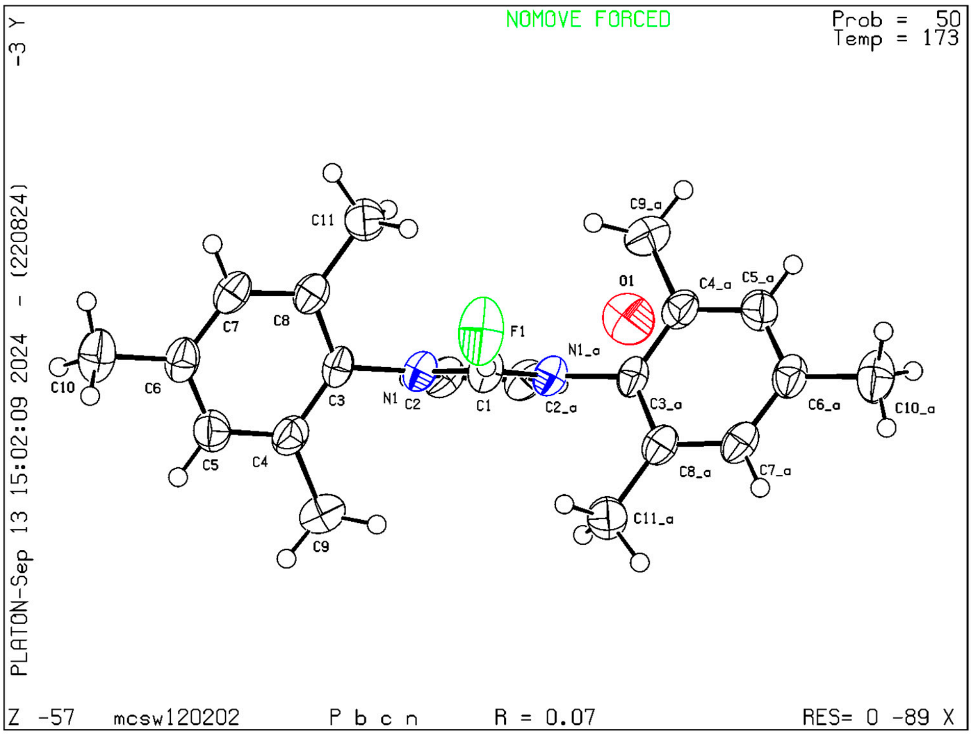

CheckCif for **1c**

Checking for embedded fcf data in CIF ...
Found embedded fcf data in CIF. Extracting fcf data from uploaded CIF, please wait

checkCIF/PLATON (basic structural check)
Structure factors have been supplied for datablock(s) **1c** mcsw120206
THIS REPORT IS FOR GUIDANCE ONLY. IF USED AS PART OF A REVIEW PROCEDURE FOR PUBLICATION, IT SHOULD NOT REPLACE THE EXPERTISE OF AN EXPERIENCED CRYSTALLOGRAPHIC REFEREE.

No syntax errors found. CIF dictionary

Please wait while processing .... Interpreting this report

Structure factor report

Datablock: 1c, mcsw120206

Bond precision:	C-C = 0.0138 A	Wavelength=0.71073	
Cell:	a=9.3091(12)	b=16.283(2)	c=10.8863(15)
	alpha=90	beta=98.592(3)	gamma=90

Temperature:173 K

	Calculated	Reported
Volume	1631.6(4)	1631.6(4)
Space group	P 21/m	P 21/m
Hall group	-P 2yb	-P 2yb
Moiety formula	C27 H37 N2, 2(C H2 Cl2), F	C27 H37 N2, 2(C H2 Cl2), F
Sum formula	C29H41Cl4FN2	C29H41Cl4FN2
Mr	578.44	578.44
Dx,g cm-3	1.177	1.177
Z	2	2
Mu (mm-1)	0.388	0.388
F000	612.0	612.0
F000'	613.37	
h,k,lmax	12,21,14	12,21,14
Nref	4129	41
Tmin,Tmax	0.911,0.933	0,638, 0,746Tmin' 0.856

Correction method= # Reported T Limits: Tmin=0.638 Tmax=0.746

AbsCorr = MULTI-SCAN

Data completeness= 0.993 Theta(max)= 28.101

R(reflections)= 0.2690(2230) wR2(reflections)= 0.6611(4100)

S = 2.545 Npar= 170

The following ALERTS were generated. Each ALERT has the format test-name_ALERT_alert-type_alert-level.

Alert level A

PLAT082_ALERT_2_A High R1 Value .... 0.27 Report
PLAT084_ALERT_3_A High wR2 Value (i.e. > 0.25) .... 0.66 Report
PLAT094_ALERT_2_A Ratio of Maximum / Minimum Residual Density .... 0.831 Report
PLAT097_ALERT_2_A Large Reported Max. (Positive) Residual Density 6.99 eA-3
PLAT934_ALERT_3_A Number of (Iobs-Icalc)/Sigma(W) > 10 Outliers .. 16 Check
0 8 0, 0 1 1,-3 4 2,-3 8 2, 1 8 2,-4 10 2, -3 3 3, 0 5 3, 0 9 3,-3 1 4,-1 1 4,-2 3 5, -5 4 5, -2 5 5, -6 1 6, -3 1 9,
PLAT971_ALERT_2_A Check Calcd Resid. Dens. 1.26Ang From F1 6.42 eA-3
PLAT971_ALERT_2_A Check Calcd Resid. Dens. 2.15Ang From F1 4.46 eA-3

All alert As can be attributed to small partly twinned crystal and solvent disorder, so far from optimal data.

Alert level B

PLAT340_ALERT_3_B Low Bond Precision on C-C Bonds .... 0.01385 Ang.

Alert level C

DIFMX02_ALERT_1_C The maximum difference density is > 0.1*ZMAX*0.75
The relevant atom site should be identified.
GOODF01_ALERT_2_C The least squares goodness of fit parameter lies
outside the range 0.80 <> 2.00 Goodness of fit given = 2.545
PLAT087_ALERT_2_C Unsatisfactory S value (Too High) .... 2.55 Check
PLAT220_ALERT_2_C NonSolvent Resd 1 C 3.5 Ratio

PLAT222_ALERT_3_C NonSolvent Resd 1 H 4.5 Ratio
PLAT230_ALERT_2_C Hirshfeld Test Diff for C9 --C11 5.2 s.u.
PLAT234_ALERT_4_C Large Hirshfeld Difference C9 --C10 . 0.20 Ang.
PLAT242_ALERT_2_C Low 'MainMol' Ueq as Compared to Neighbors of C9Check
PLAT260_ALERT_2_C Large Average Ueq of Residue Including Cl1 0.184 Check
PLAT260_ALERT_2_C Large Average Ueq of Residue Including Cl3 0.171 Check
PLAT329_ALERT_4_C Carbon Atom Hybridisation Unclear for .... C15 Check
PLAT906_ALERT_3_C Large K Value in the Analysis of Variance 70.372

And 3 other PLAT906 Alerts

More ...

PLAT911_ALERT_3_C Missing FCF Refl Between Thmin & STh/L= 0.600 7 Report
-2 0 1, 4 15 1, 4 13 2, 4 14 2, 3 15 2, 7 0 3, 3 14 3,
PLAT918_ALERT_3_C Reflection(s) with I(obs) much Smaller I(calc) . 10 Check
PLAT939_ALERT_3_C Large Value of Not (SHELXL) Weight Optimized S . 14.42 Check
PLAT971_ALERT_2_C Check Calcd Resid. Dens. 1.69 Ang From Cl3 1.92 eA-3
PLAT976_ALERT_2_C Check Calcd Resid. Dens. 0.55 Ang From C15 . -0.69 eA-3

Alert level G

PLAT002_ALERT_2_G Number of Distance or Angle Restraints on AtSite 5 Note
PLAT003_ALERT_2_G Number of Uiso or U(i,j) Restrained non-H Atoms 2 Report
PLAT171_ALERT_4_G The CIF-Embedded .res File Contains EADP Records 1 Report
PLAT172_ALERT_4_G The CIF-Embedded .res File Contains DFIX Records 3 Report
PLAT186_ALERT_4_G The CIF-Embedded .res File Contains ISOR Records 2 Report
PLAT299_ALERT_4_G Atom Site Occupancy Constrained at .... 0.5 Check
H15A H15B
PLAT304_ALERT_4_G Non-Integer Number of Atoms in ..... (Resd 2) 2.5 Check
PLAT304_ALERT_4_G Non-Integer Number of Atoms in ..... (Resd 4) 0.5 Check
PLAT411_ALERT_2_G Short Inter H...H Contact H6 .. H15B 2.01 Ang.
1-x,1/2+y,1-z = 2_656 Check
PLAT411_ALERT_2_G Short Inter H...H Contact H6 .. H15A 2.01 Ang.
1-x,1-y,1-z = 3_666 Check
PLAT432_ALERT_2_G Short Inter X...Y Contact F1 .. C1 2.77 Ang.
x,y,z = 1_555 Check
PLAT432_ALERT_2_G Short Inter X...Y Contact F1 ..C15 2.79 Ang.
-1+x,y,z = 1_455 Check
PLAT764_ALERT_4_G Overcomplete CIF Bond List Detected (Rep/Expd) . 1.11 Ratio
PLAT860_ALERT_3_G Number of Least-Squares Restraints .... 15 Note
PLAT910_ALERT_3_G Missing # of FCF Reflection(s) Below Theta(Min). 1 Note
0 0 1,
PLAT912_ALERT_4_G Missing # of FCF Reflections Above STh/L= 0.600 21 Note
PLAT913_ALERT_3_G Missing # of Very Strong Reflections in FCF .... 1 Note
-2 0 1,
PLAT941_ALERT_3_G Average HKL Measurement Multiplicity .... 1.6 Low
PLAT969_ALERT_5_G The 'Henn et al.' R-Factor-gap value .... 17.37 1 Note
Predicted wR2: Based on SigI**2 3.81 or SHELX Weight 25.97
PLAT978_ALERT_2_G Number C-C Bonds with Positive Residual Density. 1 Info

7 **ALERT level A** = Most likely a serious problem - resolve or explain
1 **ALERT level B** = A potentially serious problem, consider carefully
20 ALERT level C = Check. Ensure it is not caused by an omission or oversight
20 **ALERT level G** = General information/check it is not something unexpected

1 ALERT type 1 CIF construction/syntax error, inconsistent or missing data
21 ALERT type 2 Indicator that the structure model may be wrong or deficient
15 ALERT type 3 Indicator that the structure quality may be low
10 ALERT type 4 Improvement, methodology, query or suggestion
1 ALERT type 5 Informative message, check

It is advisable to attempt to resolve as many as possible of the alerts in all categories. Often the minor alerts point to easily fixed oversights, errors and omissions in your CIF or refinement strategy, so attention to these fine details can be worthwhile. In order to resolve some of the more serious problems it may be necessary to carry out additional measurements or structure refinements.

However, the purpose of your study may justify the reported deviations and the more serious of these should normally be commented upon in the discussion or experimental section of a paper or in the "special_details" fields of the CIF. checkCIF was carefully designed to identify outliers and unusual parameters, but every test has its limitations and alerts that are not important in a particular case may appear. Conversely, the absence of alerts does not guarantee there are no aspects of the results needing attention. It is up to the individual to critically assess their own results and, if necessary, seek expert advice.

Publication of your CIF in IUCr journals

A basic structural check has been run on your CIF. These basic checks will be run on all CIFs submitted for publication in IUCr journals (*Acta Crystallographica*, *Journal of Applied Crystallography*, *Journal of Synchrotron Radiation*); however, if you intend to submit to *Acta Crystallographica Section C* or *E* or *IUCrData*, you should make sure that full publication checks are run on the final version of your CIF prior to submission.

Publication of your CIF in other journals

Please refer to the *Notes for Authors* of the relevant journal for any special instructions relating to CIF submission.

PLATON version of 22/08/2024; check.def file version of 21/08/2024 Datablock **mcsw1c, 120206** - ellipsoid plot

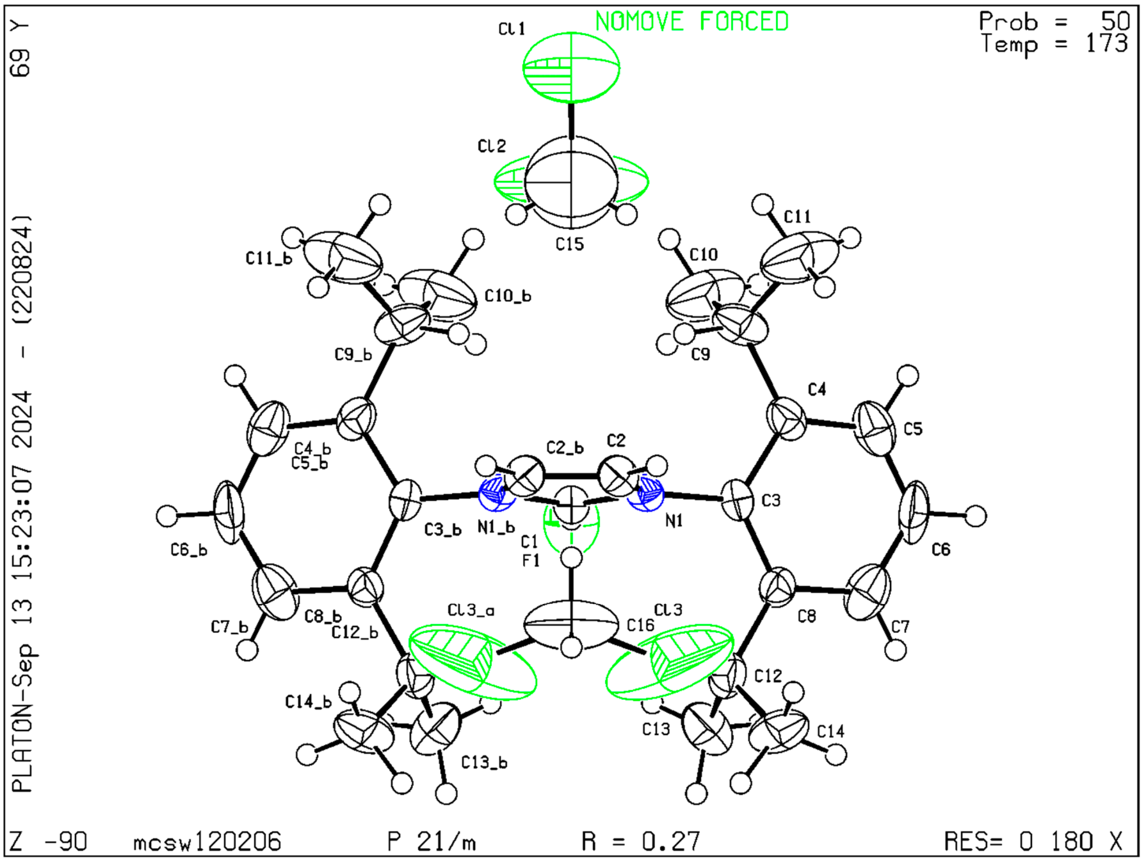

NO MOVE FORCED

CheckCif for **2**

Checking for embedded fcf data in CIF ...
Found embedded fcf data in CIF. Extracting fcf data from uploaded CIF, please wait . . . .

checkCIF/PLATON (basic structural check)

Structure factors have been supplied for datablock(s) **2**, mcsw120207

THIS REPORT IS FOR GUIDANCE ONLY. IF USED AS PART OF A REVIEW PROCEDURE FOR PUBLICATION, IT SHOULD NOT REPLACE THE EXPERTISE OF AN EXPERIENCED CRYSTALLOGRAPHIC REFEREE.

No syntax errors found. CIF dictionary
Please wait while processing .... Interpreting this report

Structure factor report

Datablock: 2, mcsw120207

Bond precision:	C-C = 0.0106 A		Wavelength=0.71073
Cell:	a=28.2902(17)	b=28.2902(17)	c=12.1774(7)
	alpha=90	beta=90	gamma=120
Temperature:	173 K		

	Calculated	Reported
Volume	8440.3(11)	8440.3(11)
Space group	R -3	R -3 :H
Hall group	-R 3	-R 3
Moiety formula	6(C15H21N4Ni), 6(F), 6(H2O), 7(O) [+ solvent]	6(C15H21N4Ni), 6(F), 6(H2O), 7(O)
Sum formula	C90 H138 F6 N24 Ni6 O13 + solvent	C90 H138 F6 N24 Ni6 O13
Mr	2230.38	2230.38
Dx,g cm-3	1.316	1.316
Z	3	3
Mu (mm-1)	1.057	1.057
F000	3516.0	3516.0
F000'	3523.34	
h,k,lmax	37,37,16	37,37,16
Nref	4617	4566
Tmin,Tmax	0.776, 0.809	0.670, 0.746
Tmin'	0.589	

Correction method= # Reported T Limits: Tmin = 0.670 Tmax = 0.746
AbsCorr = MULTI-SCAN

Data completeness= 0.989	Theta(max)= 28.147
R(reflections)= 0.0776 (2983)	wR2(reflections)= 0.2718 (4566)
S = 1.085	Npar= 214

The following ALERTS were generated. Each ALERT has the format **test-name_ALERT_alert-type_alert-level**.
Click on the hyperlinks for more details of the test.

Alert level B

PLAT245_ALERT_2_B U(iso) H1W Smaller than U(eq) O1 by 0.064 Ang** 2

PLAT245_ALERT_2_B U(iso) H2W Smaller than U(eq) O1 by 0.064 Ang** 2
PLAT306_ALERT_2_B Isolated Oxygen Atom (H-atoms Missing ?) .... O2 check
PLAT306_ALERT_2_B Isolated Oxygen Atom (H-atoms Missing ?) .... O3 check
PLAT973_ALERT_2_B Check Calcd Positive Resid. Density on Ni1 1.81 eA-3

Alert level C
PLAT084_ALERT_3_C High wR2 Value (i.e. > 0.25) .... 0.27 Report
PLAT234_ALERT_4_C Large Hirshfeld Difference C12 --C13 0.16 Ang.
PLAT260_ALERT_2_C Large Average Ueq of Residue Including F1 0.118 Check
And 3 other PLAT260 Alerts
More ...
PLAT341_ALERT_3_C Low Bond Precision on C-C Bonds .... 0.01057 Ang.
PLAT905_ALERT_3_C Negative K value in the Analysis of Variance ... -6.402
PLAT905_ALERT_3_C Negative K value in the Analysis of Variance ... -0.470
PLAT975_ALERT_2_C Check Calcd Resid. Dens. 1.00Ang From O3 0.53 eA-3
PLAT976_ALERT_2_C Check Calcd Resid. Dens. 0.89Ang From O1 -0.85 eA-3
And 2 other PLAT976 Alerts
More ...
PLAT977_ALERT_2_C Check Negative Difference Density on H1W -0.47 eA-3
PLAT977_ALERT_2_C Check Negative Difference Density on H2W -0.85 eA-3

Alert level G
PLAT007_ALERT_5_G Number of Unrefined Donor-H Atoms .... H1W H2W 2 Report
PLAT014_ALERT_1_G N.O.K. _shelx_fab_checksum Found in CIF ...... Please Check
PLAT083_ALERT_2_G SHELXL Second Parameter in WGHT Unusually Large 70.73 Why?
PLAT169_ALERT_4_G The CIF-Embedded .res File Contains AFIX 1 Recds 1 Report
PLAT304_ALERT_4_G Non-Integer Number of Atoms in ..... (Resd 4) 0.17 Check
PLAT605_ALERT_4_ Largest Solvent Accessible VOID in the Structure 240 A**3
PLAT869_ALERT_4_G Alerts related to SQUEEZE Suppressed ! Info
PLAT910_ALERT_3_G Missing # of FCF Reflection(s) Below Theta(Min). 2 Note
-1 2 0, -1 1 1,
PLAT912_ALERT_4_G Missing # of FCF Reflections Above STh/L= 0.600
PLAT969_ALERT_5_G The 'Henn et al.' R-Factor-gap value .... 9.607 Note
Predicted wR2: Based on SigI**2 2.83 or SHELX Weight 25.09
PLAT978_ALERT_2_G Number C-C Bonds with Positive Residual Density. 0 Info

0 **ALERT level A** = Most likely a serious problem - resolve or explain
5 **ALERT level B** = A potentially serious problem, consider carefully
15 **ALERT level C** = Check. Ensure it is not caused by an omission or oversight
11 **ALERT level G** = General information/check it is not something unexpected

1 ALERT type 1 CIF construction/syntax error, inconsistent or missing data
17 ALERT type 2 Indicator that the structure model may be wrong or deficient
5 ALERT type 3 Indicator that the structure quality may be low
6 ALERT type 4 Improvement, methodology, query or suggestion 2 ALERT type 5 Informative message, check

It is advisable to attempt to resolve as many as possible of the alerts in all categories. Often the minor alerts point to easily fixed oversights, errors and omissions in your CIF or refinement strategy, so attention to these fine details can be worthwhile. In order to resolve some of the more serious problems it may be necessary to carry out additional measurements or structure refinements. However, the purpose of your study may justify the reported deviations and the more serious of these should normally be commented upon in the discussion or experimental section of a paper or in the "special_details" fields of the CIF. checkCIF was carefully designed to identify outliers and unusual parameters, but every test has its limitations and alerts that are not important in a particular case may appear. Conversely, the absence of alerts does not guarantee there are no aspects of the results needing attention. It is up to the individual to critically assess their own results and, if necessary, seek expert advice.

Publication of your CIF in IUCr journals
A basic structural check has been run on your CIF. These basic checks will be run on all CIFs submitted for publication in IUCr journals (*Acta Crystallographica*, *Journal of Applied Crystallography*, *Journal of Synchrotron Radiation*); however, if you intend to submit to *Acta Crystallographica Section C* or *E* or *IUCrData*, you should make sure that full publication checks are run on the final version of your CIF prior to submission.

Publication of your CIF in other journals
Please refer to the *Notes for Authors* of the relevant journal for any special instructions relating to CIF submission.

PLATON version of 22/08/2024; check.def file version of 21/08/2024

Datablock 2, mcsw120207 - ellipsoid plot

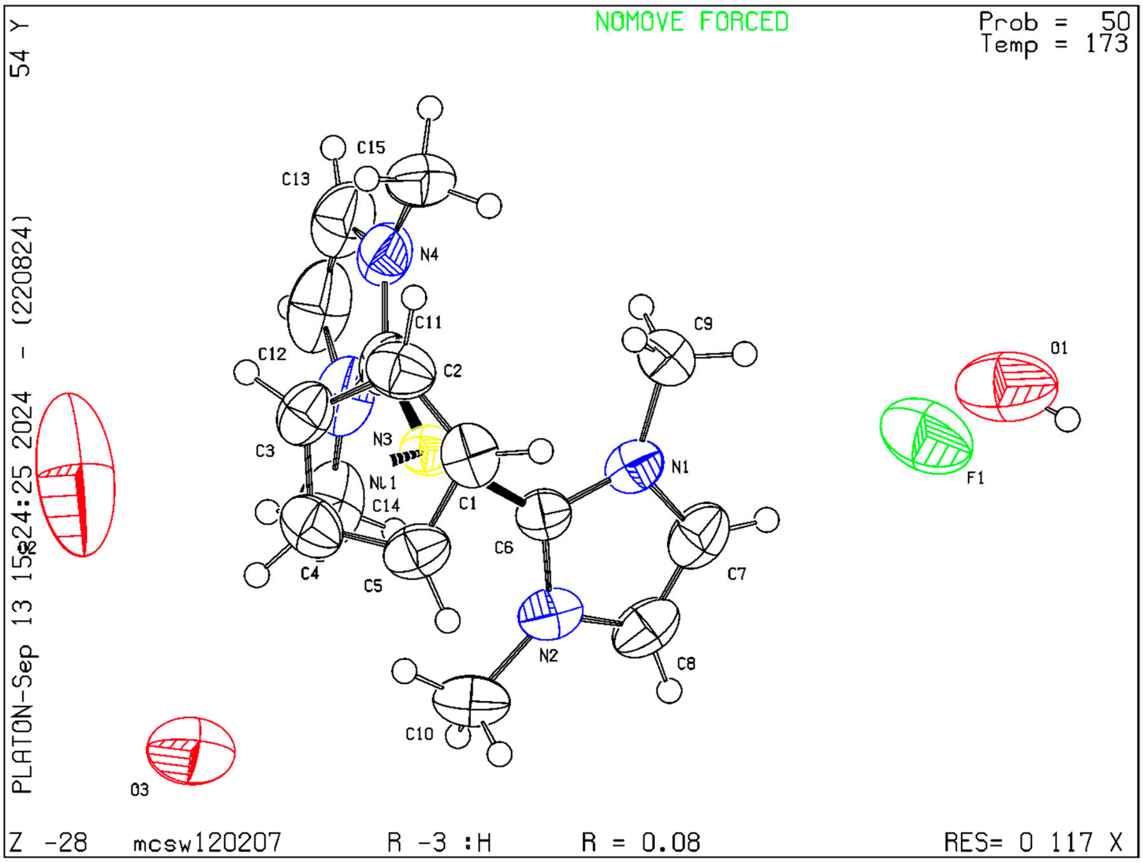

NO MOVE FORCED

CheckCif for **3**

CheckCIF (basic structural check) running

Checking for embedded fcf data in CIF ...
Found embedded fcf data in CIF. Extracting fcf data from uploaded CIF, please wait **. . . .**

checkCIF/PLATON (basic structural check)

Structure factors have been supplied for datablock(s) **3a,** mcsw111207

,REPORT IS FOR GUIDANCE ONLY. IF USED AS PART OF A REVIEW PROCEDURE FOR PUBLICATION, IT SHOULD NOT REPLACE THE EXPERTISE OF AN EXPERIENCED CRYSTALLOGRAPHIC REFEREE.

No syntax errors found. CIF dictionary
Please wait while processing .... Interpreting this report

Structure factor report

Datablock: 3a, mcsw111207

Bond precision:	C-C = 0.0088 A	Wavelength=0.71073	
Cell:	a = 20.7771(10)	b = 20.7771(10)	c=16.3363(8
	alpha = 90	beta = 90	gamma=120
Temperature: 173 K			

	Calculated	Reported
Volume	6107.4(7)	6107.4(7)
Space group	P -3 c 1	P -3 c 1
Hall group	-P 3 2"c	-P 3 2"c
Moiety formula C	C42 H48 F2 N4 Ni [+ solvent]	C42 H48 F2 N4 Ni
Sum formula	C42 H48 F2 N4 Ni [+ solvent]	C42 H48 F2 N4 Ni
Mr	705.55	705.55
Dx,g cm-3	1.151	1.151
Z	6	6

Mu (mm-1)	0.517	0.517
F000	2244.0	2244.0
F000'	2246.80	
h,k,lmax	28,28,22	28,28,22
Nref	5529	5444
Tmin,Tmax	0.883,0.925	0.820, 0.927

Tmin' 0.813

Correction method= # Reported T Limits: Tmin = 0.820 Tmax = 0.927

AbsCorr = MULTI-SCAN

Data completeness= 0.985	Theta(max)= 29.185
R(reflections)= 0.1018 (3180)	wR2(reflections)= 0.2192 (5444)
S = 1.142	Npar= 228

The following ALERTS were generated. Each ALERT has the format **test-name_ALERT_alert-type_alert-level**.
Click on the hyperlinks for more details of the test.

Alert level B

RINTA01_ALERT_3_B The value of Rint is greater than 0.18
Rint given 0.218

PLAT020_ALERT_3_B The Value of Rint is Greater Than 0.12 .... 0.218 Report

Alert level C

PLAT094_ALERT_2_C Ratio of Maximum / Minimum Residual Density .... 2.49 Report
PLAT220_ALERT_2_C NonSolvent Resd 1 C Ueq(max)/Ueq(min) Range 5.3 Ratio
PLAT222_ALERT_3_C NonSolvent Resd 1 H Uiso(max)/Uiso(min) Range 5.2 Ratio
PLAT250_ALERT_2_C Large U3/U1 Ratio for <U(i,j)> Tensor(Resd 1) 2.8 Note
PLAT341_ALERT_3_C Low Bond Precision on C-C Bonds .... 0.000884 Ang.
PLAT906_ALERT_3_C Large K Value in the Analysis of Variance ...... 11.417 Check
PLAT906_ALERT_3_C Large K Value in the Analysis of Variance ...... 4.126 Check
PLAT911_ALERT_3_C Missing FCF Refl Between Thmin & STh/L= 0.600 68 Report
-2 4 0, -3 8 0, -2 9 0, -1 9 0, -4 10 0, -3 10 0, -3 11 0, -5 13 0, -2 4 1, -1 4 1, -5 6 1, -6 7 1, -4 7 1, -7 8 1, -8 9 1, -1 9 1, -10 11 1,-3 11 1, -1 11 1, -8 14 1, 0 0 2, 0 1 2, -2 4 2, -3 5 2, -2 5 2, -3 6 2, -8 10 2, -6 11 2, -3 11 2,-10 12 2, -7 13 2, -5 14 2, -1 2 3, -2 3 3, -3 5 3, -4 7 3, -6 8 3, -4 8 3, -8 9 3, -8 12 3, -7 12 3, -4 13 3, -4 8 4, -3 9 4, -6 7 5, -4 8 5, -7 9 5, -9 11 5, 0 0 6, -3 5 6,-5 8 6, -9 13 7, -11 15 7, 0 2 8, -2 3 8, -8, 10 8, -11 13 8,-10 11 9, 0 2 10, -2 3 10, -1 3 10, -8 9 10, -10 12 10, -9 12 10, -9 13 10, -8 11 11, -8 12 11, -8 9 12,

Alert level G

PLAT019_ALERT_1_G _diffrn_measured_fraction_theta_full/*_max < 1.0 0.997 Report
PLAT066_ALERT_1_G Predicted and Reported Tmin&Tmax Range Identical ? Check
PLAT605_ALERT_4_G Largest Solvent Accessible VOID in the Structure 2 73 A** 3
PLAT869_ALERT_4_G ALERTS Related to the Use of SQUEEZE Suppressed ! Info
PLAT910_ALERT_3_G Missing # of FCF Reflection(s) Below Theta(Min). 1 Note
0 1 0,
PLAT912_ALERT_4_G Missing # of FCF Reflections Above STh/L= 0.600 20 Note
PLAT913_ALERT_3_G Missing # of Very Strong Reflections in FCF··· 0 0 2 1 Note
PLAT969_ALERT_5_G The 'Henn et al.' R-Factor-gap value .... 2.601 Note
Predicted wR2: Based on SigI**2 8.43 or SHELX Weight 19.20
PLAT978_ALERT_2_G Number C-C Bonds with Positive Residual Density. 9 Info

0 **ALERT level A** = Most likely a serious problem - resolve or explain
2 **ALERT level B** = A potentially serious problem, consider carefully
8 **ALERT level C** = Check. Ensure it is not caused by an omission or oversight
9 **ALERT level G** = General information/check it is not something unexpected

2 ALERT type 1 CIF construction/syntax error, inconsistent or missing data
4 ALERT type 2 Indicator that the structure model may be wrong or deficient
9 ALERT type 3 Indicator that the structure quality may be low
3 ALERT type 4 Improvement, methodology, query or suggestion
1 ALERT type 5 Informative message, check.

It is advisable to attempt to resolve as many as possible of the alerts in all categories. Often the minor alerts point to easily fixed oversights, errors and omissions in your CIF or refinement strategy, so attention to these fine details can be worthwhile. In order to resolve some of the more serious problems it may be necessary to carry out additional measurements or structure refinements. However, the purpose of your study may justify the reported deviations and the more serious of these should normally be commented upon in the discussion or experimental section of a paper or in the "special_details" fields of the CIF. checkCIF was carefully designed to identify outliers and unusual parameters, but every test has its limitations and
alerts that are not important in a particular case may appear. Conversely, the absence of alerts does not guarantee there are no aspects of the results needing attention. It is up to the individual to critically assess their own results and, if necessary, seek expert advice.

Publication of your CIF in IUCr journals

A basic structural check has been run on your CIF. These basic checks will be run on all CIFs submitted for publication in IUCr journals (*Acta Crystallographica*, *Journal of Applied Crystallography*, *Journal of Synchrotron Radiation*); however, if you intend to submit to *Acta Crystallographica Section C* or *E* or *IUCrData*, you should make sure that full publication checks are run on the final version of your CIF prior to submission.

Publication of your CIF in other journals
Please refer to the *Notes for Authors* of the relevant journal for any special instructions relating to CIF submission.

PLATON version of 22/08/2024; check.def file version of 21/08/2024 Datablock 3amcsw111207 - ellipsoid plot

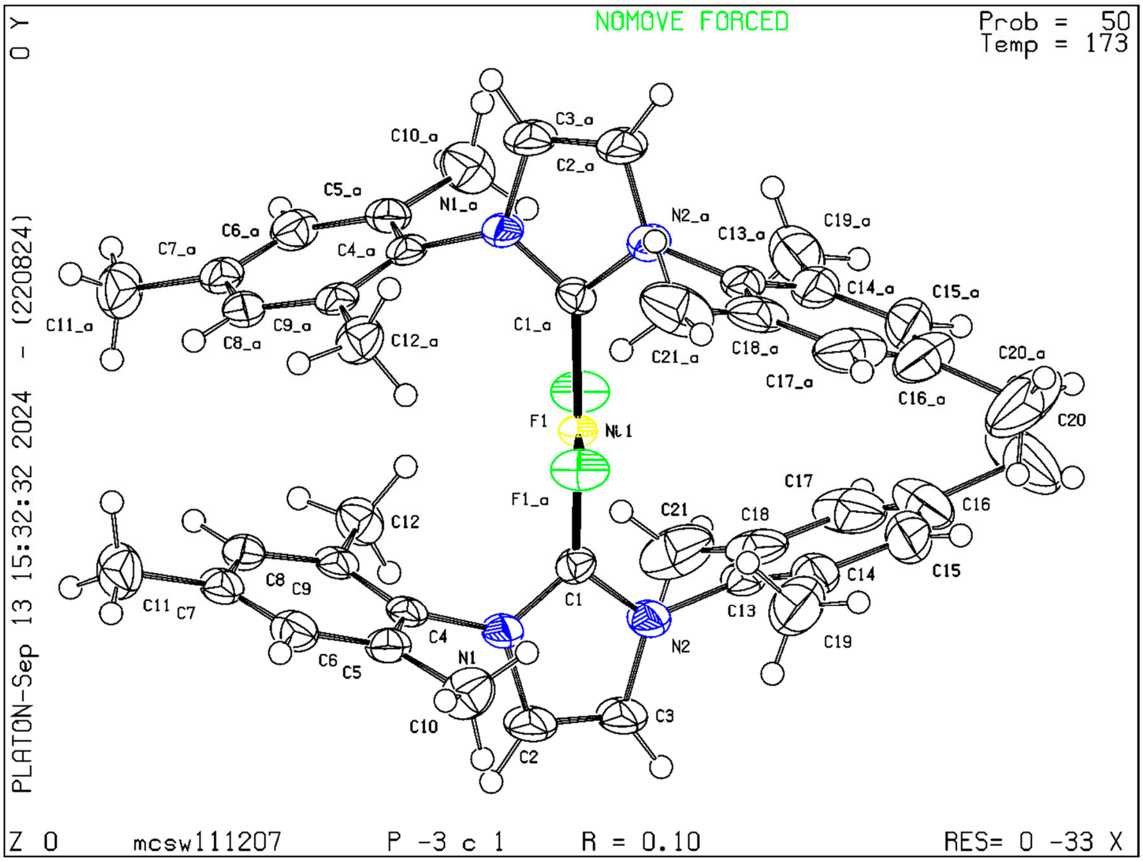

NO MOVE FORCED
